# Comparing machine learning and deep learning approaches to predicting the seismic response of slab-column connections

**DOI:** 10.1038/s41598-026-50962-9

**Published:** 2026-05-11

**Authors:** Mahmoud A. El-Mandouh, Hassan Youssef, M. S. Elborlsy, Mostafa A. Ebied

**Affiliations:** 1 Civil Engineering Department, Faculty of Engineering, Beni-Suef University, Beni-Suef, 62511 Egypt; 2 Civil Construction Technology Department, Faculty of Technology and Education, Beni-Suef University, Beni-Suef, 62511 Egypt; 3 Department of Process Control Technology, Faculty of Technology and Education, Beni-Suef University, Beni-Suef, Egypt; 4 Department of Electronics Technology, Faculty of Technology and Education, Beni-Suef University, Beni-Suef, Egypt

**Keywords:** Structural engineering, Punching moment prediction, Drift ratio estimation, Machine learning, Deep learning, Hybrid model, CNN-LSTM, Gradient boosting, Random forest, XGBoost algorithm, Engineering, Mathematics and computing

## Abstract

Slab-column joints constitute the most sensitive elements of flat plate structures during seismic forces since they are likely to experience brittle failure. This paper examines the predictive accuracy of an extensive Machine Learning (ML) and Deep Learning (DL) solutions for predicting seismic performance of slab-column connections, such as punching moment (M) and Drift Ratio (dr). The ML models under study are Ridge Regression (RR), Linear Regression (LR), Lasso Regression (Lasso R), Elastic Net (EN), Support Vector Regression (SVR), Gradient Boosting (GB), random Forest (RF), and Extreme Gradient Boosting (XGBoost). The models of DL that were analyzed are Long Short-Term Memory (LSTM), Convolutional Neural Network (CNN), Recurrent Neural Network (RNN), and a hybrid version that incorporates CNN and LSTM (CNN-LSTM). The analysis shows that in (M) prediction, the performance of GB was the best with a coefficient of determination (R^2^) of 0.870850, a Root Mean Square Error (RMSE) of 0.378246, and a Mean Absolute Error (MAE) of 0.282686. In the DL models, CNN had the best accuracy using R^2^ of 0.827864, MAE of 0.335228, and RMSE of 0.436680. RF was better than other ML models in dr prediction with an R^2^ of 0.565014, MAE of 0.440262, and RMSE of 0.619478. Once again, CNN performed better than the rest of the DL models, with an R^2^ of 0.470568, MAE of 0.513140, and RMSE of 0.683429.

## Introduction

When assessing and designing RC structures to behave in seismic performance, the accurate estimation of the (M and dr) is used to assess local and global parameters of response. Punching shear at slab-column connections is a brittle mode of failure that can result in disproportionate collapse when total lateral displacements are restricted. Conversely, the dr becomes a major parameter of global deformation demand that is directly linked with the damage states, ductility capacity, and serviceability limits. The two (M and dr) are very nonlinear and interdependent and depend on a variety of factors such as slab thickness, column size, axial load percentage, slab reinforcement ratios, material strengths, and the cyclic loading properties^1–3^. This complexity in itself presents serious difficulties to traditional methods of analysis and empirical studies, and international standards such as ACI 318-25^4^, CSA A23.3-24^5^, NZS 3101-06^6^, and ECP 203-2020^7^ follow different formulations and assumptions to approximate such parameters. The classic analytical and code-based tools may be based on simplistic assumptions or empirical correlations that are obtained using a small amount of experimental data, and thus, they may cause great discrepancy when used with complicated structural arrangements or atypical loading conditions^8^. With the help of ML and DL, Artificial Intelligence (AI) will be capable of reaching these constraints and achieve much more accurate and reliable predictions of (M and dr) due to learning the complex nonlinear relationships. AI is changing the engineering field with data-driven decisions, automation, and predictive modeling to develop more intelligent, efficient, and sustainable engineering systems. AI, which is defined as machine simulation of human intelligence, improves the decision-making process, resource optimization, and the solution of complex, non-linear, and ill-posed problems with high fault tolerance and can learn by example^9,10^. At present, many aspects of civil infrastructure are planned, operated, and controlled by the use of AI by civil engineers, and its particular applications to other subfields of civil engineering^11^. ML is a subfield of AI that deals with computer programs that are learned and become better with time and experience. It develops models based on training data in order to produce predictions or decisions without using explicit programming. ML offers solutions that are quick and efficient in modeling complex systems and find application in a very diverse range of applications^12–15^. DL, another sub-domain in terms of ML, builds on multi-layered neural networks that mirror the human brain, and can automatically derive the complex features and provide high-quality accuracy in every application, such as natural language processing, image recognition, and prediction^16–18^.

The paper is designed in the following way: Section “Literature review” will summarize the corresponding literature, section “Methodology” will present the methodology of the research that includes the description of the system studied, the formulation of the problem, and the framework of the model adopted. These involve the preparation of the data, the selection of the features, as well as the use of both traditional and neural network models to predict (M and dr) in RC slab column connection. Section “Results and discussion” includes the results presentation and analysis, providing extensive evaluation metrics like R^2^, MAE, and RMSE, and is complemented by comparative tables and visualization that help to outline the performance of both ML and DL models. Lastly, section “Conclusion” is the conclusion of the study where the key findings of the study are summarized, and their implications on the structural engineering practice are discussed, and future research directions are given.

## Literature review

Performance-based seismic design focuses on accurately predicting structural behavior across various earthquake scenarios, aiming for improved post-earthquake functionality. It requires identifying damage stages to guide efficient retrofitting or cost-effective design through hysteretic response prediction. These studies consistently show that ML/DL models, particularly ensemble and hybrid approaches, can achieve superior accuracy compared to existing design codes and physics-based equations. However, a common limitation across many existing works is their narrow focus on predicting a single, specific structural response, often utilizing a limited suite of algorithms without comprehensive benchmarking against a broad range of modern ML and DL architectures for multiple critical engineering outputs. Table 1 presents a comprehensive analysis of existing studies on ML/DL applications for predicting structural responses in RC systems.

**Table 1:** Summary of Existing Studies on ML/DL Applications for Predicting Structural Responses in RC Systems

Ref	Objective(s)	Result(s)	Limitation(s)
^19^	Predict the PHL of RC columns using an AdaBoost ensemble model	The AdaBoost model significantly outperformed existing empirical formulas and base ML models (ANN, SVM, CART), achieving high accuracy (e.g., MAPE of 6.74% vs. 30.39% for the best empirical model)	The model is limited to predicting a single structural parameter, PHL Trained on a relatively small dataset (133 samples), which may restrict its generalizability to other structural responses or more complex datasets
^20^	Develop a hybrid metaheuristic LS-SVM model (LS-SVMR-CSA) to predict the lateral strength of RC columns, applicable to flexure-, shear-, and flexure-shear-critical types	The RBF kernel-based LS-SVMR-CSA model outperformed other ML models (MARS, RF) and traditional physics-based equations, achieving high accuracy (e.g., R^2^ = 0.98) across all column failure types	The model specializes in a single output (lateral strength) and relies on a complex Hybrid metaheuristic optimization for tuning, which may be computationally intensive Less accessible compared to standard, out-of-the-box ML models
^21^	Predict the transverse reinforcement ratio of RC columns using ML to improve on empirical models	The XGBoost model achieved the best performance (R^2^ = 0.873, MAE = 0.161) Model interpreted via SHAP/PDP, showing alignment with mechanical laws	Limited to a single structural parameter (transverse reinforcement) Uses a single best-performing ML model (XGBoost) lacking a comprehensive comparative analysis of a wide range of ML and DL models for different structural responses
^22^	To forecast the punching shear strength of concrete slabs (reinforced with CFRP, GFRP, or steel) using five ML algorithms and compare their performance against existing empirical code equations	SVR was the best-performing model, achieving an R^2^ of 96.23% for GFRP-reinforced slabs ML models significantly outperformed traditional code equations (e.g., ACI 440), which were found to be overly conservative	Only tested five ML models (MLR, Bagging-DT, RF, SVR, XGBoost) No DL Models: Did not investigate any DL architectures Focused solely on punching shear strength, not other structural responses like moment or drift
^23^	Develop XGBoost model for predicting shear strength of PC beams and derive reliability-based reduction factors for code-compliant design	XGBoost model achieved high accuracy (R^2^ = 0.98) and outperformed design codes & FE analysis SHAP provided global & local explainability Code-compliant reduction factors were derived	Limited to a single ML model (XGBoost) and a single structural problem (shear in PC beams) Does not provide a comprehensive comparison across a wide range of ML and DL models for different structural responses
^24^	To review recent applications of ML in the design, construction, and inspection of RC bridges, focusing on structural design, quality management, and health monitoring	ML and DL methods show promise in tasks such as strength prediction, damage detection, crack identification, and scour depth estimation Hybrid and ensemble models often outperform single models	Limited ML use in design; models lack generalizability Relies on U.S.-centric data (e.g., NBI), reducing global relevance DL models are data-hungry and noise-sensitive No broad comparison of ML/DL models for key responses like (M and dr)
^25^	To predict the MIDR and the Median of IDA Curves (M-IDAs) for seismic performance assessment of RC MRFs using various ML models	ANN and XGBoost were the best models, achieving high R^2^ values for predicting MIDR and M-IDAs A GUI was developed for practical seismic limit-state capacity estimation	Excludes key DL models (e.g., CNN, LSTM) Predicts only seismic responses (drift/acceleration) Not punching shear Lacks broad ML/DL comparative benchmarking
^26^	To use ML for a binary classification task (risky vs. not risky) of existing RC buildings based on field-collected structural data	RF achieved 100% accuracy in classifying building risk A sensitivity analysis identified building age, stirrup spacing, concrete strength, steel yield strength, and corrosion as the most critical factors	Uses binary classification (risky/not risky) instead of predicting quantitative engineering Predicts only binary risk, not key engineering parameters Uses limited ML models, omitting advanced ensembles and DL 100% accuracy implies possible data leakage, reducing real-world reliability
^27^	To build ML models for classifying various seismic damage states in RC wall structures and identify the best-performing algorithm	XGBoost and CatBoost were the top-performing models, achieving 88% accuracy in classifying three damage states (DS1, DS2, DS3) based on the MIDR A GUI was developed for practical use	Focuses on RC wall damage classification, excluding other systems and continuous outputs Uses homogeneous spectral inputs, missing key material/section details Lacks comparison with linear ML models and all DL architectures
^28^	To design ML models for forecasting the continuous MIDR of RC wall structures	The DNN model achieved the highest accuracy (R^2^ = 0.9177), outperforming Random Forest (RF, R^2^ = 0.8984) and XGBoost (R^2^ = 0.8889) A GUI was developed based on the DNN model	Evaluates only three models (RF, XGBoost, DNN), omitting a wider suite of ML/DL algorithms Focuses solely on global drift in RC walls, excluding key responses like punching shear Rely on seismic-specific inputs (spectral data), limiting applicability to datasets with standard material/geometric properties
^29^	To predict seismic acceleration responses of precast segmental self-centering CFST bridges using ML models (Conv1D-LSTM, XGBoost, RFR), validated against shaking table and finite element data	Conv1D-LSTM outperformed XGBoost and RFR with R^2^ = 0.9643 for acceleration response prediction The fiber finite element model exhibited close correlation with the experimental findings (errors < 10%)	Predicts only acceleration, not drift or moment Tested only on time-series data, not tabular data Lacks comparison with most DL models and broader ML benchmarks Small dataset (116 samples) limits generalizability
^30^	To develop an explainable ML model using XGBoost to predict the drift capacity of RC walls with special boundary elements, and to compare its performance against the empirical equation in ACI 318 − 19	The XGBoost model significantly outperformed the ACI 318 − 19 equation, with R^2^ = 0.87 and RMSE = 0.23% SHAP analysis provided interpretability, identifying λb​, shear stress, and reinforcement configuration as key features	Predicts only drift in RC walls, omitting key responses Small dataset (*n* = 164) limits generalizability Excludes DL models and relies solely on XGBoost Uses post-hoc (SHAP) interpretation instead of transparent modeling
^31^	To develop a DL-based visual inspection system using CNN and ANN for automated defect detection, classification, and severity prediction in RC bridge substructures	The system achieved accuracies of 90.4% for defect detection, 81% for defect classification (cracking, erosion, honeycomb, scaling, spalling), and 78% for binary severity prediction (severe/non-severe)	Focuses only on image-based defect severity (binary), not structural responses like drift or moment Uses a small, imbalanced dataset (*n* = 180 images) Exclusively employs DL (CNN/ANN), omitting traditional ML benchmarks Inapplicable to numerical/tabular engineering data
^32^	To review and analyze the current state of using ML techniques for predicting the behavior of various FRP-strengthened RC members (beams, columns, slabs, joints, and bond), identify research gaps, and assess the efficacy of ML compared to empirical methods	ML models significantly outperform existing empirical design guidelines in prediction accuracy for all FRP-strengthened members Supervised learning (e.g., XGBoost, ANN, RF) is the most favored method. Ensemble and hybrid models often show superior performance 32% of studies focused on beams, 32% on columns, 22% on bond strength, 6.5% on slabs, 6.5% on materials, and only 1% on beam-column joints	The review is qualitative and lacks quantitative performance metrics (e.g., R^2^, MAE, RMSE) for direct model comparisons It excludes DL architectures entirely and offers minimal analysis of critical responses like dr Its broad scope across applications prevents deep, performance-focused evaluation of specific predictive tasks
^33^	To develop a novel ML-integrated MSA procedure for efficiently generating seismic fragility curves of RC wall structures, reducing computational cost vs. traditional MSA	RF model achieved best performance (accuracy = 0.99, F1-score = 0.98). The proposed ML-MSA procedure produced fragility curves closely matching those from full nonlinear analysis	Limited to one EDP-IM pair (MIDR-PGA) Performance degrades at high MIDR thresholds due to data scarcity No DL models were explored or compared
^34^	To develop a hybrid data-driven method that integrates ML with a physics-based hysteretic model to predict the full seismic response history (hysteretic behavior) of RC columns efficiently and accurately	Outperformed classical fiber-based models in predicting hysteretic curves, backbone parameters, and energy dissipation for RC columns Achieved higher R^2^, lower errors (RMSE/MAE), and reduced computational time by 99.7% (4s vs. 1268s)	The model relies on a predefined hysteretic shape, lacks full data-driven flexibility, and depends on manual feature selection It predicts only global force-displacement, not local responses like moment or shear, limiting detailed design use
^35^	To develop an ML model for the concurrent prediction of 3D building-level characteristics and the applicability of pushover analysis for masonry-infilled RC buildings, accounting for infill wall effects	The CatBoost algorithm achieved exceptional performance on the test set: R^2^ = 0.977 for torsional irregularity, R^2^ = 0.997 for fundamental period, R^2^ = 0.923 for MPMR, and a 98.6% accuracy for POA applicability It significantly outperformed DNN, RF, AdaBoost, and DT models	The model predicts only global building properties (e.g., period) and cannot assess local responses (e.g., punching, drift), which are crucial for detailed design It specializes in specific seismic parameters, not general structural responses Its accuracy relies on synthetic data, with no validation on real-world experimental datasets
^36^	To predict the flexural capacity of ECC-strengthened RC beams using a Physics-Informed ML framework that integrates empirical knowledge	The proposed EGML model showed slightly lower accuracy (RMSE = 0.101) than a pure data-driven model (RMSE = 0.091) but demonstrated superior physical consistency and generalization	Narrow scope (only flexural capacity). Small dataset (*n* = 194) Complex, computationally expensive framework Highly dependent on prior empirical knowledge
^37^	To forecast the failure modes and punching shear capacity of shear-reinforced slab–column connections using hybrid ML models (GWO/WOA-XGBoost)	XGBoost achieved the highest accuracy (81.8%) for failure modes classification WOA-XGBoost performed best for resistance prediction (R^2^ = 0.9642, RMSE = 0.126 MN) SHAP analysis identified key influential parameters	Narrow focus on one element (slab-columns) Complex hybrid models are computationally heavy Tied to a small, specific dataset (*n* = 328), limiting generalizability to other responses or elements
^38^	To forecast the compressive strength of steel fiber–reinforced concrete using six ML models (SVR, GPR, RFR, XGBR, ANN, KNN) and identify the best performer	Gaussian Process Regression was the best model (R^2^ = 0.93, RMSE = 16.54) It excelled at capturing complex nonlinearities and peak behaviors (e.g., optimal w/c ratio) Fiber content was the most important feature. ANN and KNN performed poorly	Only predicts compressive strength with traditional ML, excluding DL and key responses like drift or moment No hybrid ML-DL exploration Static training and single-parameter validation lack robustness
^39^	Aimed at creating a DNN model to forecast the thickness and length of RC shear walls from architectural plans, accelerating the initial structural design phase	A DNN model achieved extremely high predictive accuracy for wall dimensions, with an R^2^ of 0.995 for thickness and 0.994 for length The model was trained on an extensive, feature-engineered dataset from 165 buildings	The model only predicts wall dimensions, not key structural responses like punching or drifting It requires architectural plans as input and was not compared to simpler, more efficient ML models
^40^	To improve seismic assessment of slab-column connections using ML-driven regression and a new DNN-based constitutive model for FEA, augmented with GANs	The DNN model significantly improved hysteretic response prediction in FEA and outperformed existing design codes in accuracy	Focuses on simulation enhancement, not broad benchmarking of ML/DL models for predictive accuracy on key structural responses
^41^	To forecast the punching shear capacity of FRP–RC slabs using ML models and compare their accuracy against existing empirical models and design codes	The Adaptive Boosting (AdaBoost) model achieved accuracy (R^2^ = 0.99, RMSE = 29.83, MAE = 23.00), outperforming six empirical models and codes SHAP analysis provided interpretability of input feature importance	The study is limited to FRP slabs and does not evaluate a wide range of advanced ML/DL models (e.g., XGBoost, CNN, LSTM) or extend predictions to key seismic responses like drift capacity—a focus of our work

- **Research gap**
  The review of the literature revealed several notable gaps and limitations, which can be outlined as follows:
  - Most of the studies involve a limited number of ML (e.g., XGBoost, RF) or single DL (ANN, CNN), as well as no general comparisons.
  - There are no direct comparisons of ML and DL methods.
  - Both the moment of punching (M and dr) are seldom predicted simultaneously.
  - ML-based features used in hybrid DL models (e.g., CNN-LSTM) are hardly investigated.
  - Many datasets are either small or very specific, which reduces the ability to generalize.
  - Assessment is a common practice that only uses R^2^ without using other metrics, such as MAE and RMSE, regularly.
  - DL is primarily utilized on images, as opposed to tabular data, to predict structural responses.
- **Contributions**
  According to the above analysis, the field and key contributions of the study are outlined:
  - Offers the most detailed assessment of both ML (8 models) and DL (4 models, including a hybrid CNN-LSTM) of two important responses (M and dr) in RC systems, the widest comparison of the two so far.
  - The study also predicts (M and dr) concurrently, unlike the previous studies, providing a wider structural performance outlook.
  - Multiple and powerful performance indices (R^2^, MAE, RMSE) are used to provide a fair and transparent comparison of the models.
  - Demonstrates the decisive advantage of tree-based ML algorithms (GB, XGBoost, RF) over linear and DL on the structured engineering data, which was not previously mentioned in previous works.
  - Upon analyzing tabular engineering data, not only image data is assessed in terms of DL architectures (CNN, RNN, LSTM, CNN-LSTM), but also highlights weaknesses and possibilities.
  - Hybrid DL models (CNN-LSTM) structural-response prediction tests and reports, which are practically absent in the literature.
  - Regarding all the models that were assessed to predict (M), GB was the best model with the highest R^2^ of 0.870850, (MAE) of 0.282686, and (RMSE) of 0.378246. XGBoost was the next with the R^2^ of 0.857051, MAE of 0.313696, and RMSE of 0.397941. RF too had great predictive accuracy with the R^2^ value of 0.840906.
  - RF in predicting dr was superior to other ML models and was able to produce the highest R^2^ of 0.565014, MAE of 0.440262, and RMSE of 0.619478.
  - Offers evidence-based suggestions that can be used when predicting structural response using ML or DL, for future research and industry use.

## Methodology

**Fig. 1:**
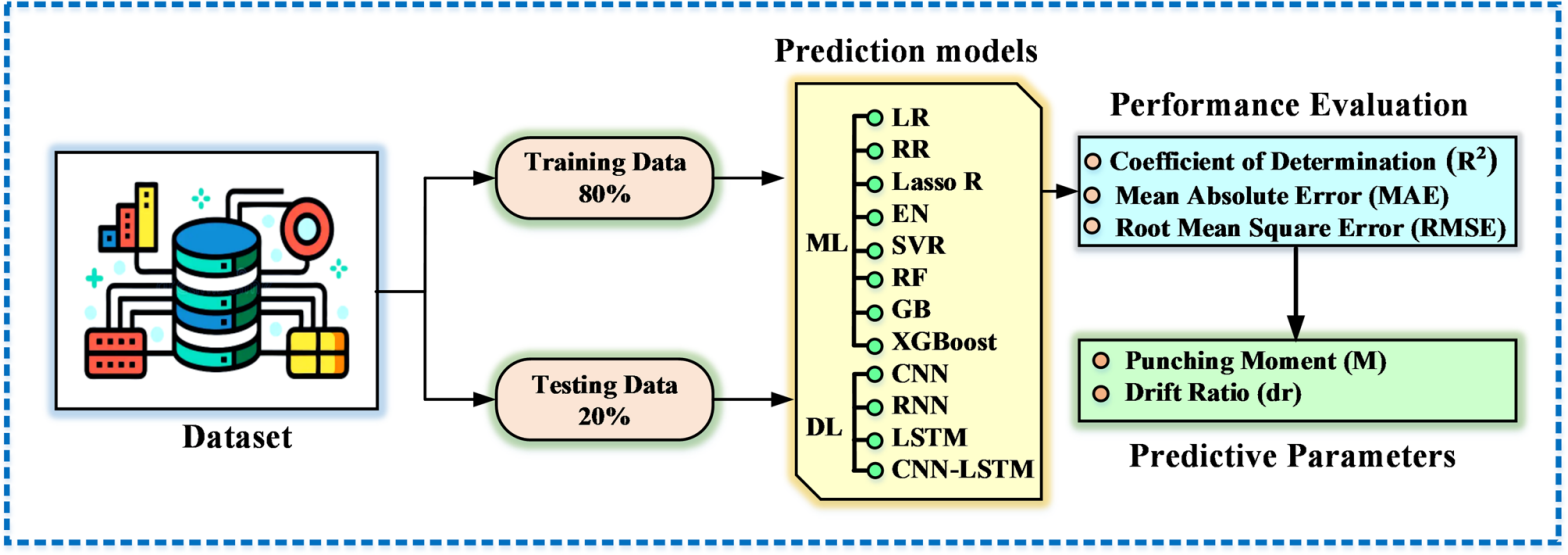
Methodology and model performance evaluation workflow

**Fig. 2:**
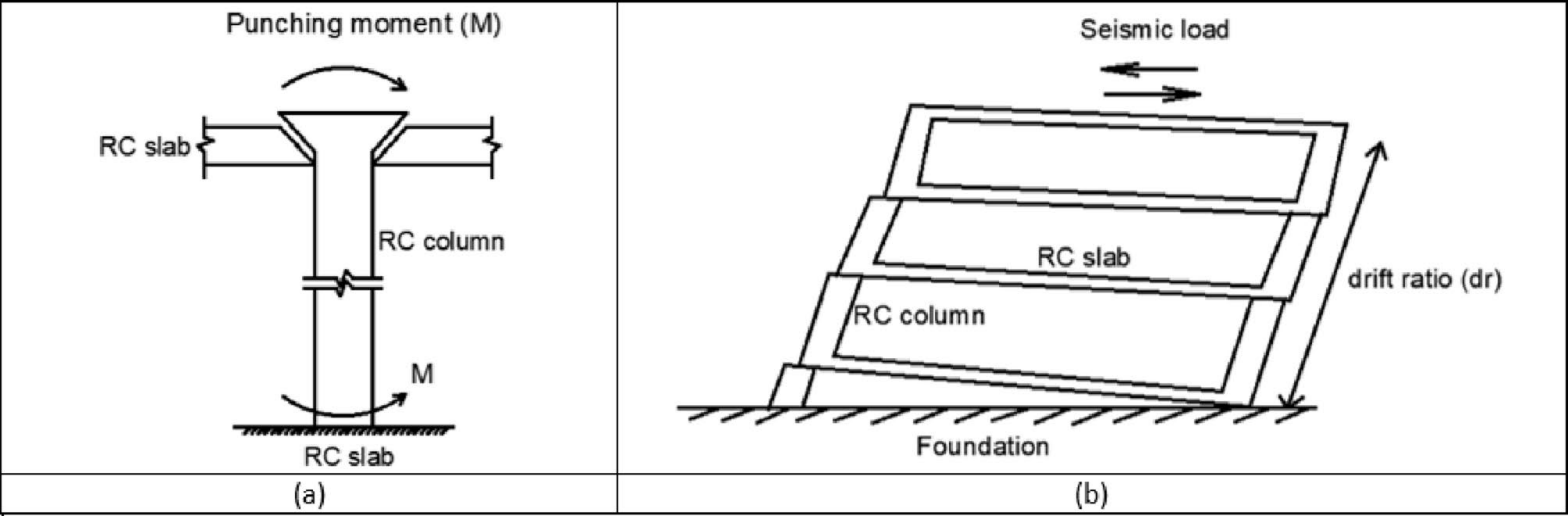
Schematic illustration of the assessed response parameters: (**a**) punching moment (M) and (**b**) drift ratio (dr)

The methodological framework employed in this research is depicted in Fig. 1, beginning with data collection, utilizing key geometric and material properties of concrete slabs as input features. This data was then used to train and evaluate a comprehensive suite of both ML (8 models) and DL (4 models, including a hybrid CNN-LSTM). To ensure a comprehensive and fair evaluation, the selected models were chosen to represent different methodological categories, including linear models (LR, RR, Lasso, EN), ensemble models (RF, GB, XGBoost), and deep learning architectures (CNN, RNN, LSTM, CNN-LSTM). This selection enables a systematic benchmarking framework rather than arbitrary model inclusion and aligns to compare different approaches for predicting (M) and (dr). The performance of each model was rigorously assessed using standard regression metrics, including (R^2^, MAE, RMSE), to identify the most accurate and reliable predictive model. The overall workflow is summarized in Fig. 1. The workflow explicitly links data preprocessing, model training, and performance evaluation to the main objective of predicting M and dr and comparing different modeling approaches. To improve clarity and avoid ambiguity, a schematic figure was added to illustrate the two main response parameters investigated in this study, M and dr. Figure 2 clarifies the physical meaning of M as a local response parameter associated with slab–column connection behavior, and dr as a global deformation parameter representing the lateral response of the system under seismic loading. In this paper, “punching moment” means the unbalanced moment transferred to the column from the slab at the slab-column joint at the time of punching shear failure. In reinforced concrete flat slab structures under lateral or seismic loading, the unbalanced moment, together with the gravity-induced axial force in the column, causes shear stresses on the critical section around the column. If these stresses exceed the punching shear capacity of the slab, a punching shear failure occurs.

### Data overview and visualization

**Fig. 3:**
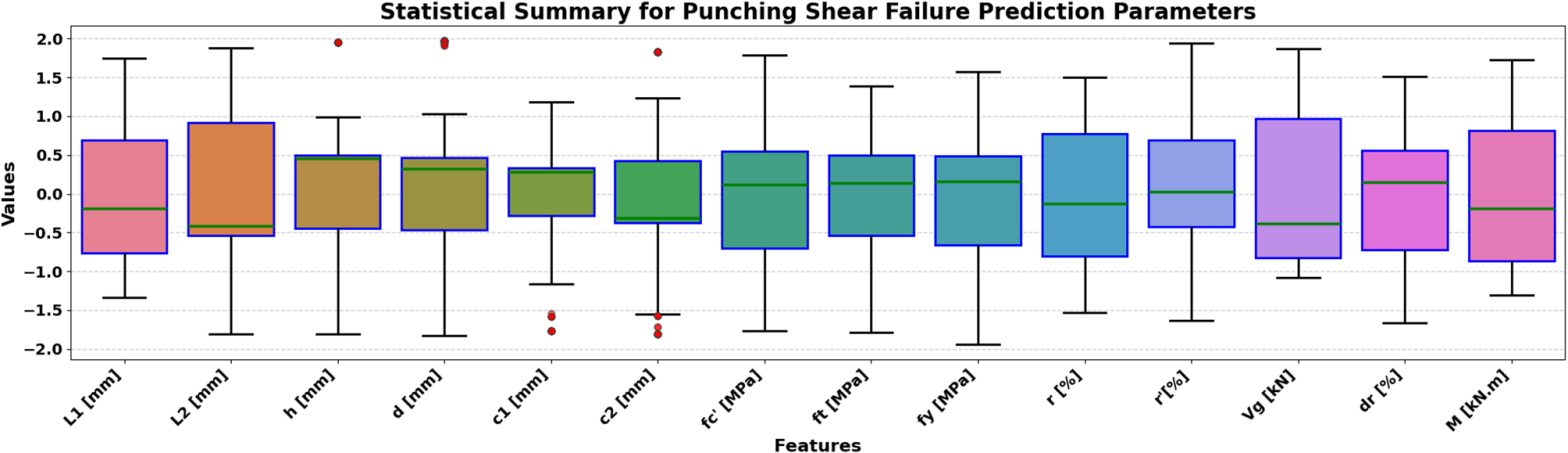
Comparative analysis of input parameters influencing punching shear failure prediction accuracy. Boxes represent the interquartile range (25th–75th percentiles), the horizontal line within each box indicates the median, and the whiskers denote the data range excluding outliers. Negative values indicate the response direction according to the adopted sign convention and do not imply unphysical behavior

**Fig. 4:**
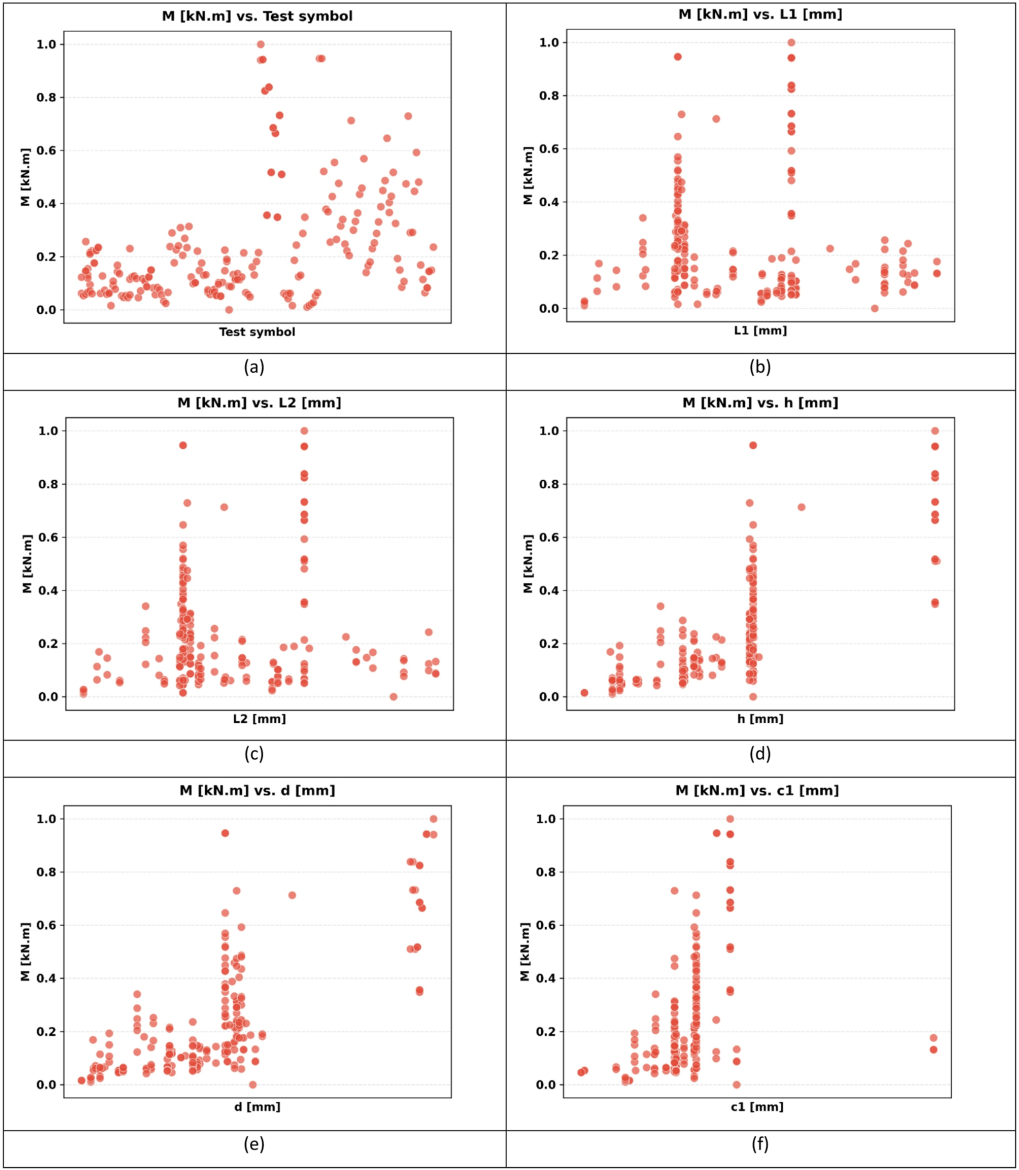

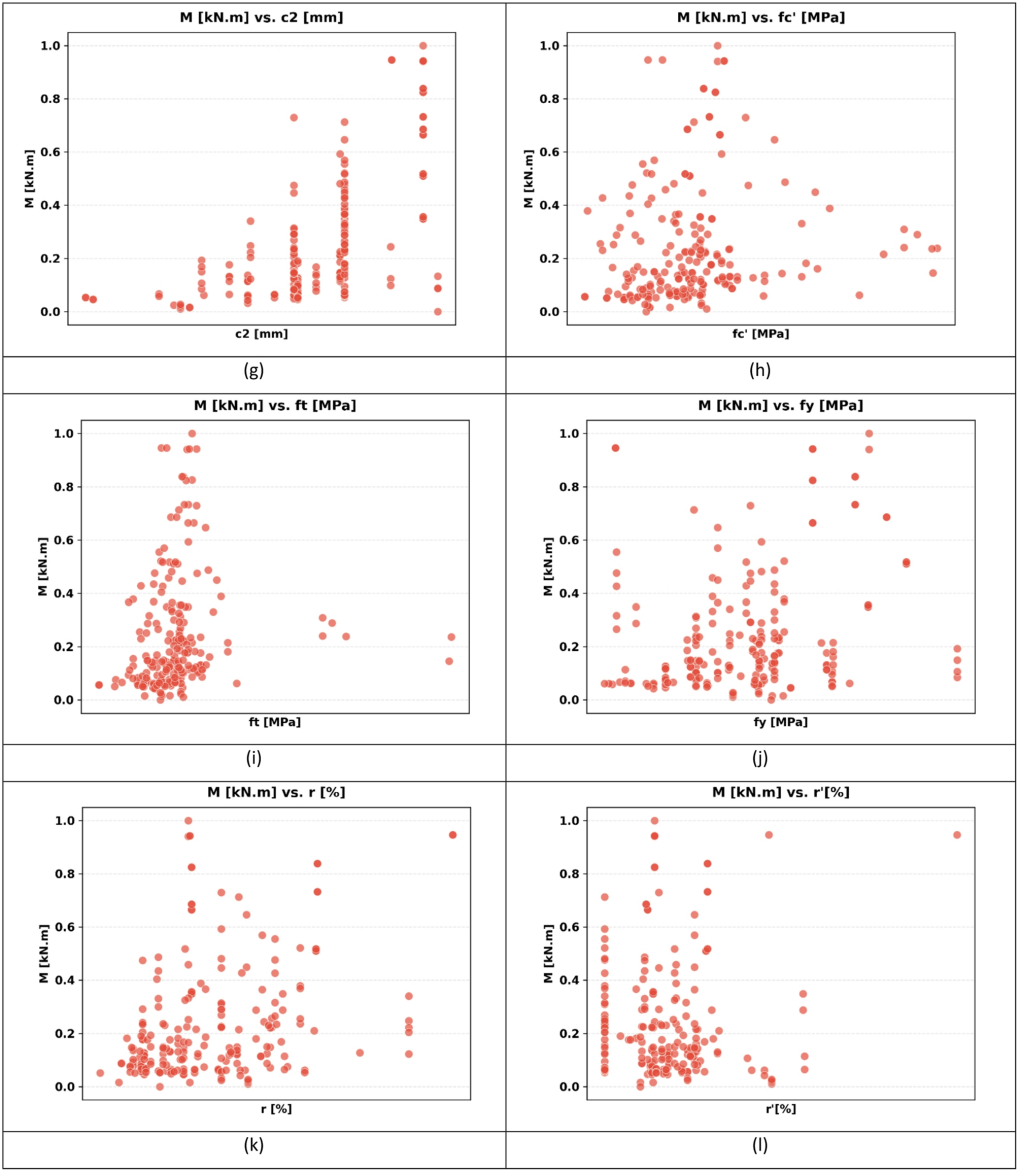

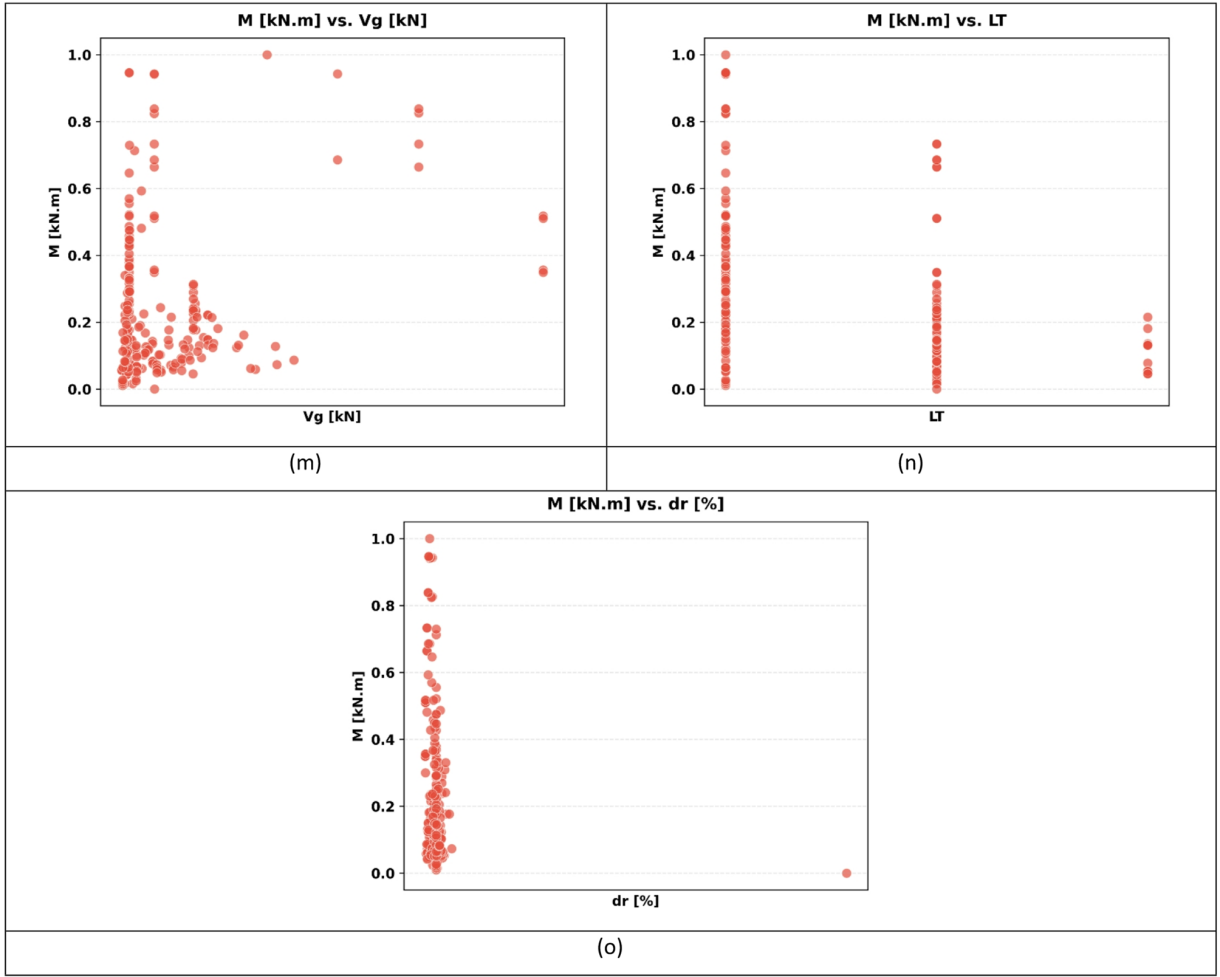
Influence of structural parameters on punching shear resistance: a multi-variable visual analysis: (**a**) variation of punching moment with test type, (**b**) slab length parallel to loading (L1), (**c**) slab length normal to loading (L2), (**d**) slab thickness (h), (**e**) effective depth (d), (**f**) column side parallel to loading (c1), (**g**) column side normal to loading (c2), (**h**) compressive strength of concrete (f′c), (**i**) tensile strength of concrete (ft), (**j**) yield strength of reinforcement (fy), (**k**) flexural reinforcement ratio (ρ), (**l**) compressive reinforcement ratio (ρ′) ,(**m**) punching moment vs. shear force (Vg), (**n**) punching moment vs. load transfer (LT), and (**o**) M vs. dr

**Fig. 5:**
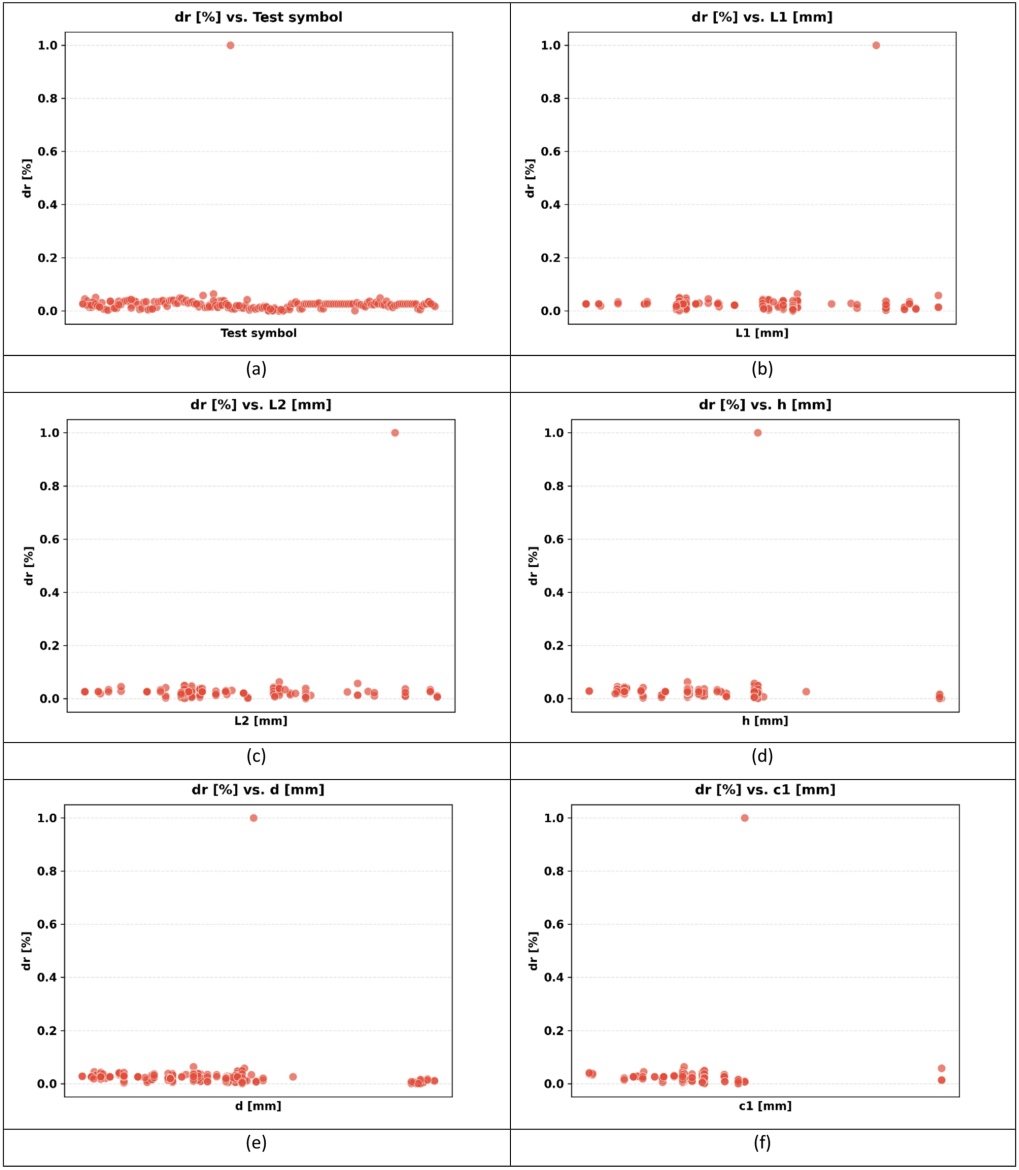

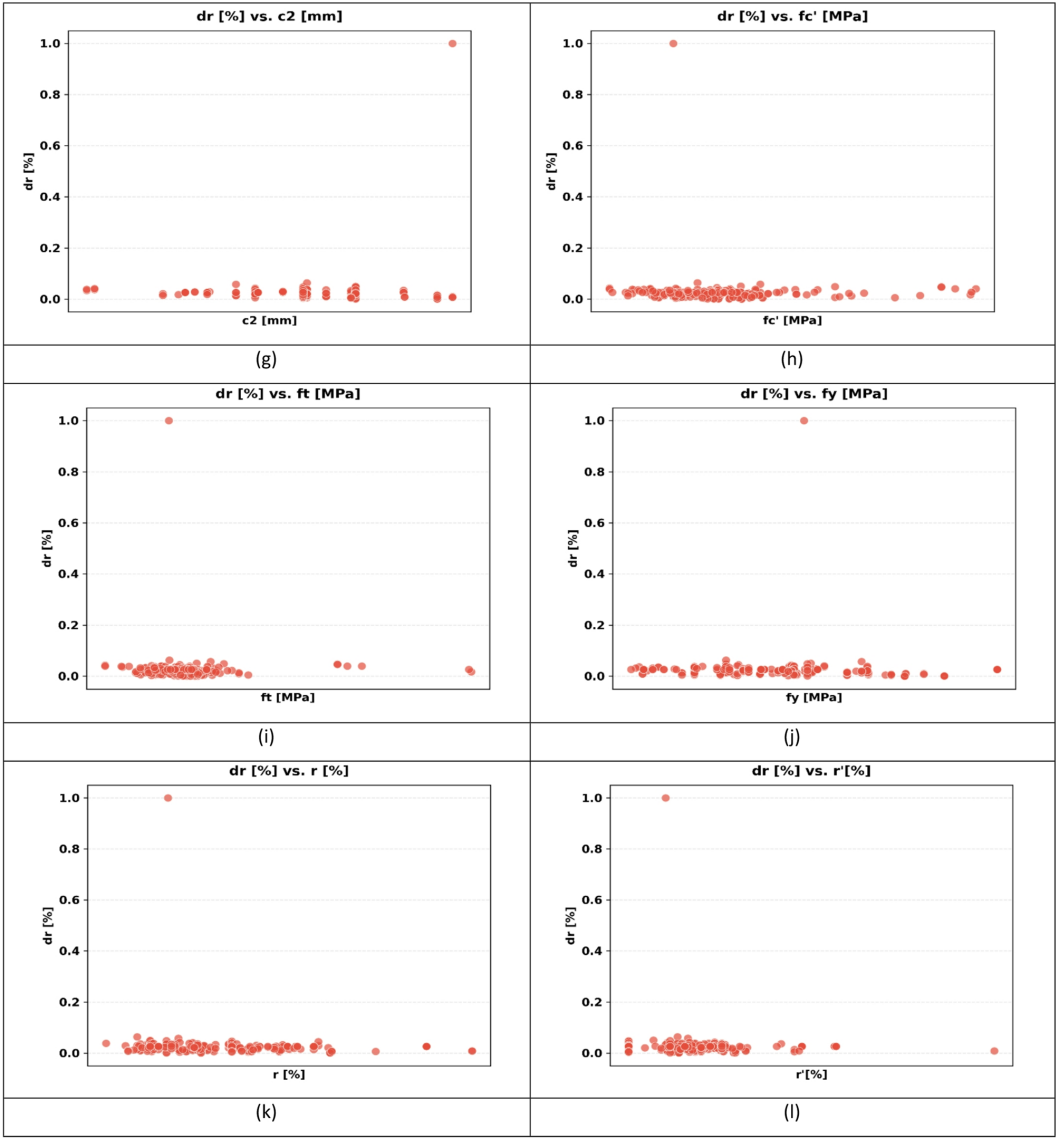

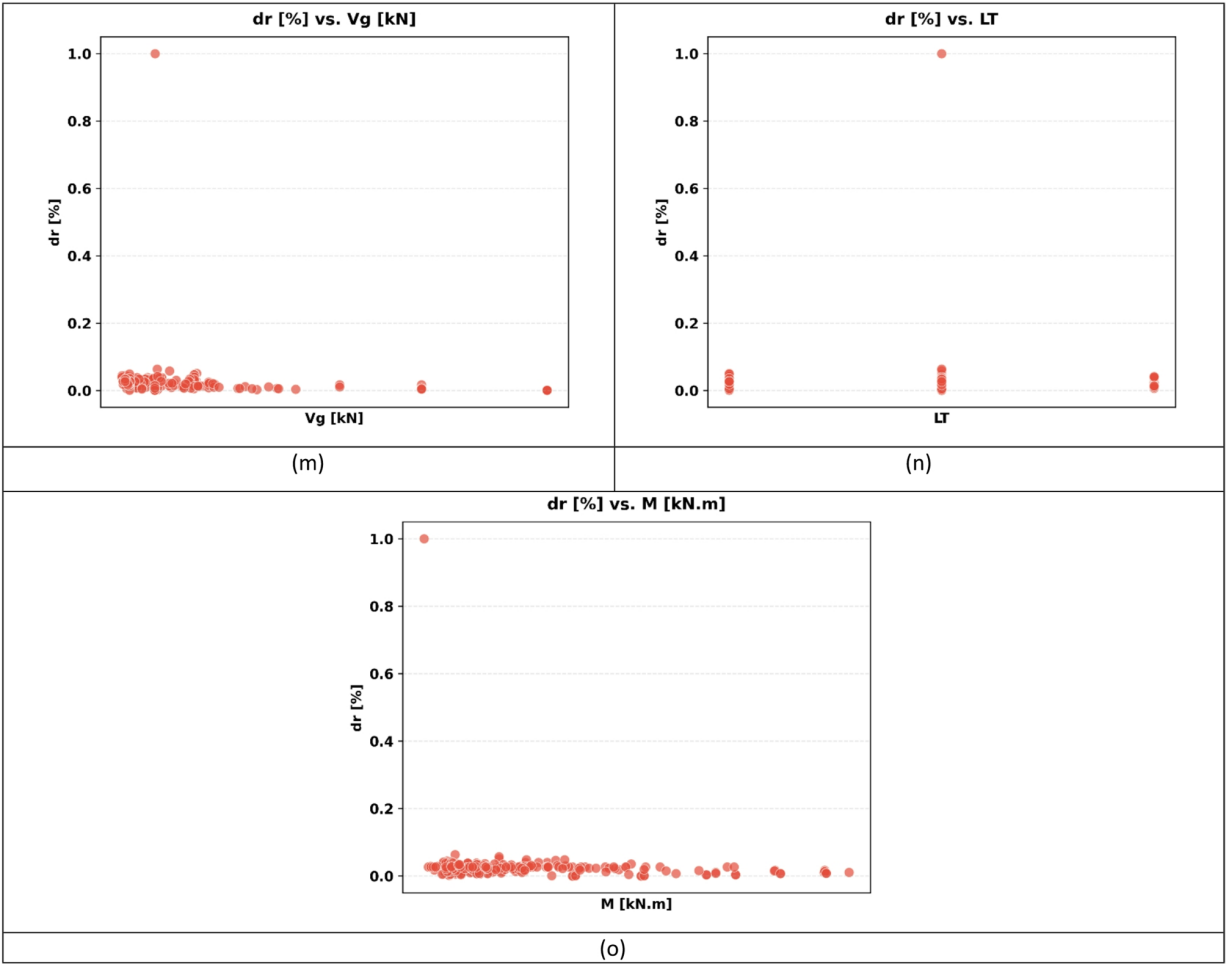
Influence of structural parameters on dr: a multi-variable visual analysis: (**a**) test type, (**b**) slab length parallel to loading (L_1_), (**c**) slab length normal to loading (L_2_), (**d**) slab thickness (h), (**e**) effective depth (d), (**f**) column side parallel to loading (c_1_), (**g**) Column side normal to loading (c_2_), (**h**) Compressive strength of concrete (f’c), (**i**) Tensile strength of concrete (f_t_), (**j**) Yield strength of reinforcement (f_γ_), (**k**) Flexural reinforcement ratio (ρ), (**l**) Compressive reinforcement ratio (ρ’), (**m**) Punching moment vs. shear force (Vg), (**n**) Punching moment vs. load transfer (LT), and (**o**) M vs. dr

**Fig. 6:**
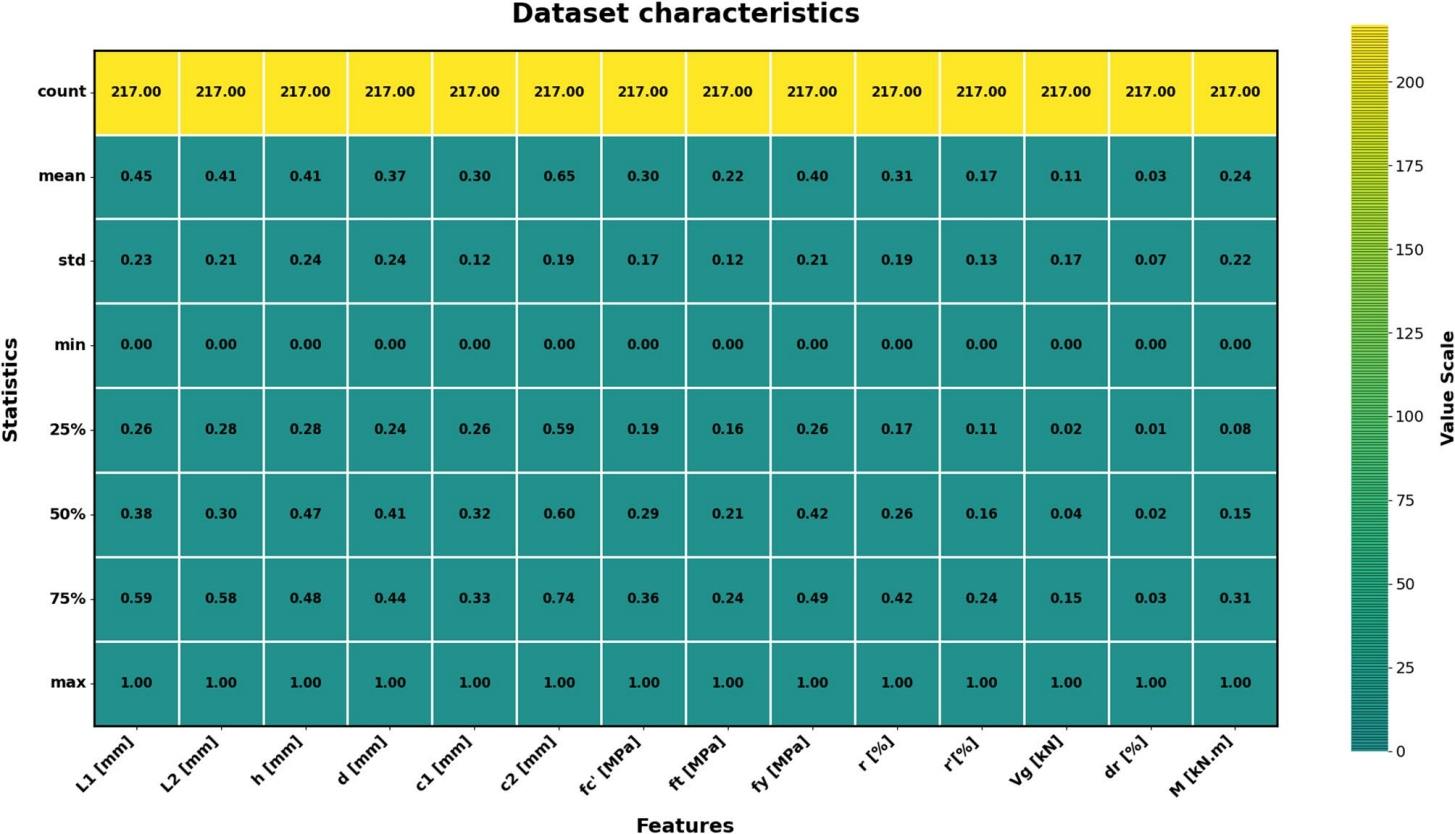
Feature variability analysis for predictive modeling in structural engineering

The sample used in the paper has 217 records, each having a group of variables that can be used to predict the seismic behaviour of slab-column connections. These records were compiled from published experimental studies, and only specimens with sufficiently complete geometric, material, reinforcement, loading, and response information were included in the final database. A complete list of source studies is provided in the revised manuscript and Supplementary Data. The dataset was divided into 80% for training and 20% for testing, and this split was performed randomly at the individual specimen level over the complete dataset of 217 records, rather than at the study level or test-series level, and all model evaluations were performed on unseen test data to ensure generalization. Figure 3 shows the overall statistics of the main variables to predict the punching shear failure of RC slabs. In Fig. 3, boxplots are interpreted such that the colored boxes represent the interquartile range, the central horizontal line denotes the median, and the whiskers indicate the spread of the data excluding outliers. Negative values reflect the sign convention of the recorded response and represent the opposite response direction rather than invalid physical behavior. These variables are geometric dimensions, material properties, reinforcement properties, and loading conditions. The geometric parameters include L1 and L2 parallel and normal to the loading direction, respectively, slab thickness (h), effective depth (d), and column side lengths (c1, c2). The concrete compressive strength (f’c), tensile strength (ft), and reinforcement yield strength (fy) are some of the material properties that are crucial in determining the mechanical properties of the slab in different load conditions. The flexural ratio (ρ) and compressive ratio (ρ−) of the reinforcement system define the serious influences on the flexural, ductile, and failure behavior of the structural component. Applied gravity load (Vg) indicates the initial vertical loading before lateral loading, and the loading type (LT) indicates the character of the lateral loading, which can be either monotonic or uniaxial cyclic, or even biaxial cyclic. Concerning the output parameters, the dataset will contain the dr, which is the deformation ability of the slab, and the moment at (M), which is the measure of the structural resistance of the ultimate state. Figure 4 introduces a multi-variable analysis of the structural parameters that control the punching shear resistance in RC slabs that incorporates 15 important relationships in a single visual structure. Figure 4a defines the baseline of correlation between (M) and test type because protocol-dependent failure mechanisms are revealed. Figure 4b and c measure the modulating role of slab geometry length parallel and normal to loading (L1) and (L2) in distributing loads. Figure 4d and e indicate the decisive roles played by slab thickness (h) and effective depth (d) in strengthening the sectional rigidity, and Fig. 4f and g reveal that shear forces are focused by the size of columns (c1 parallel to loading, and c2 normal to loading). Moving on to material properties, Fig. 4h and i decipher contributions to the strength of concrete (f′c and f_t_), and Fig. 4j correlates the reinforcement yield strength (f) to the post-cracking resilience. Figure 4k and l unwind the optimum values of flexural (ρ) and compressive (ρ′) reinforcement ratio in terms of controlling cracks. This analysis leads to a dynamic interaction: the interrelations between Fig. 4m determine moment deformation limits (M) and shear force (Vg) and Fig. 4n superimposes the moment and load transfer (LT) and defines the redistribution of forces when deforming, creating a force redistribution pathway; finally, Fig. 4o plots moment and dr, determining deformation ability. All these panels taken together split up how the interdependence of geometric design, material choice, and load-response relationships determines shear failure, which gives engineers a comprehensive package of tools to draw upon in the prevention of punching shear collapse by designing their buildings optimally in advance. Figure 5 systematically examines the effects of structural parameters on dr in RC slabs; the 15 paramount relationships are combined into one diagnostic structure. The apparent outlier was retained because it corresponds to a valid experimental observation in the compiled dataset and reflects the natural variability of structural response rather than a data error. Its inclusion allows the analysis to better represent realistic response dispersion and to evaluate the robustness of the predictive models under non-uniform data conditions. Figure 5(a) plots dr versus test type and shows that deformation patterns depend on protocol: Fig. 5b and c measure the dependence of deformation distribution on slab geometry length parallel to loading (L_1_) and normal to loading (L_2_). Figure 5d and e illustrate the determining factors of slab thickness (h) and effective depth (d) in regulating the stiffness degradation, and the effect of column dimensions (c1 loading parallel and c2 loading normal) on localized defor_2_mation is revealed in Fig. 5f and g. Moving on to material properties, Fig. 5h and i decode the contribution to the crack propagation resistance by the concrete strengths (f [ and f [ ] ), and Fig. 5j relates the yield strength of reinforcement (f) to the ductility of deformation. Figure 5k and l unveil the capacity of flexural (r) and compressive (r) reinforcement ratio to reduce excessive drifts. The analysis culminates in critical interactions: Fig. 5m correlates (M) with shear force (Vg) to define deformation thresholds; Fig. 5n maps moment against load transfer (LT), exposing deformation redistribution mechanisms; and Fig. 5o ties moment to dr, quantifying collapse progression. Collectively, these panels dissect how geometric configuration, material selection, and force-transfer mechanisms dictate deformation behavior, providing a diagnostic framework for optimizing seismic resilience and serviceability against progressive collapse. Using the analysis of Fig. 6, one can see all the statistical peculiarities of a dataset comprising 217 records and 14 different features, and have the important information to perform a predictive model. The analysis shows that there is a great heterogeneity among variables, with twelve features having great dispersion with standard deviations ranging between 0.17 and 0.24, indicating complex underlying relationships of variables, whereas only two features have low dispersion, with standard deviations of 0.07 and 0.11, indicating deterministic underlying patterns. Extreme value distributions are present by having strong minimum to maximum ranges of 0.00 to 1.00 between all features, and the quartile analysis confirms asymmetric data distributions, with the most significant being in features whose quartile values of between 0.42 and 0.74 are significantly higher than the 0.26 to 0.47 median. The variability profile presented in this case requires strong ML methods since it may have high dispersion values, including a mean of 0.03 and a standard deviation of 0.07, and low variance parameters with a mean of 0.65 and a standard deviation of 0.19, as noted in the variability profile, necessitating regularization methods to reduce noise sensitivity but capture nonlinear relationships. This preprocessing is especially important when dealing with datasets of moderate size, where it becomes possible to efficiently train the models and not lose enough complexity to be able to detect patterns in predictive systems. In Figs. 4 and 5 and a data point located on or near the zero axis does not imply that the corresponding variable does not influence M or dr. Instead, it indicates that, for that specific sample or within that local feature range, the model-estimated contribution is approximately neutral relative to the combined effects of the other input variables.

### ML algorithms

**Fig. 7:**
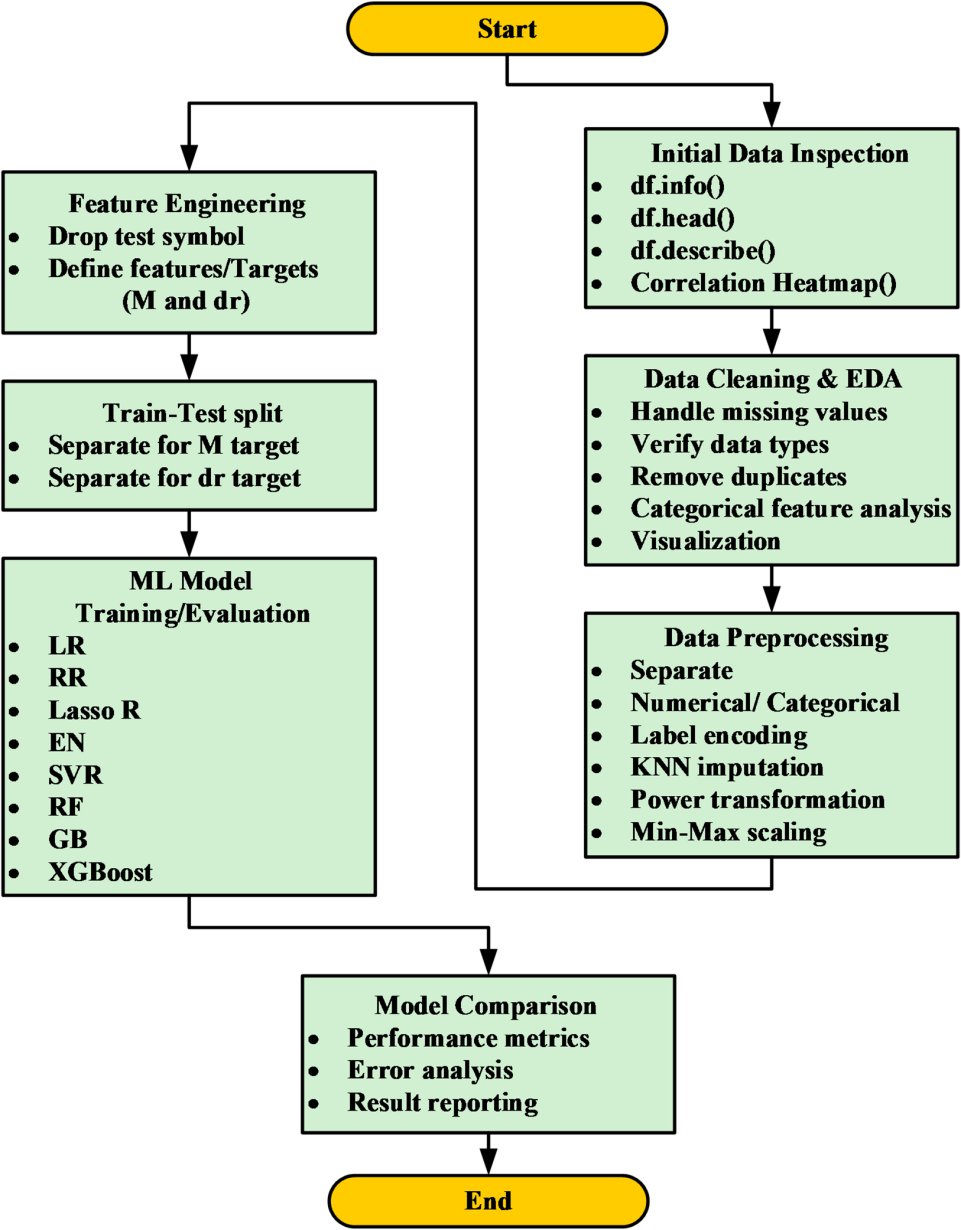
Sequential ML framework for predicting (M and dr ) in RC systems

Figure 7 shows a workflow architecture of machine learning to predict punching moment (M and dr). It starts with a stage of exploratory analysis based on heatmaps and statistics to reveal the feature relationships and anomalies. Data cleaning implies KNN imputation, duplicate and type correction. Label-encoded categorical features are used, and through power transformation and min-max scaling, normalized continuous variables. In the feature engineering process, non-informative data (e.g., test symbols) are eliminated, and feature predictors based on the domain are introduced. M and dr prediction are then split on the dataset. Eight linear (LR, RR, Lasso R, EN, SVR) and ensemble (RF, GB, XGBoost) models are trained to predict geometry, material characteristics, and reinforcement information to structural responses. Hyperparameter tuning based on cross-validation guarantees that the model applies to general data, whereas assessment based on experimental data available as holdouts of the dataset determines predictive ability in terms of error measures and residuals. The predictive piping on ML models on punching moments ( M and dr) in a benchmark of an effective workflow in structural vulnerability assessment. The pipeline is a standard that can be replicated and enables performance-based design through the determination of parameterized failure thresholds. Table 2 concludes the efficacy of models as they are valuable in the improvement of the structural performance of slab-column connections under seismic load.

**Table 2:** Comparative analysis of ML algorithms for structural performance prediction

Algorithm	Category	Model complexity	Treatment of features	Transparency	Algorithm
LR	Statistical Approach	Low Complexity & Interpretable	Requires Normalization/Feature Scaling	Low	LR
RR	Regularized linear model	Low complexity with L2 regularization	Requires feature scaling	Low	RR
Lasso R	Regularized linear model	Low complexity with L1 regularization	Requires feature scaling	Low	Lasso R
EN	Regularized linear model	Low complexity with combined regularization	Requires feature scaling	Low	EN
SVR	Kernel-based model	Moderate complexity, kernel-dependent	Requires feature scaling	Moderate	SVR
RF	Ensemble method	Moderate, scales with tree count	Handles raw features well	Moderate	RF
GB	Ensemble method (boosting)	Moderate, sequential tree optimization	Handles heterogeneous features	Moderate	GB
XGBoost	Gradient boosting framework	Moderate Complexity, Performance-Optimized	Automatic Feature Handling; Robust to Missing Values	Moderate	XGBoost

### DL algorithms

**Fig. 8:**
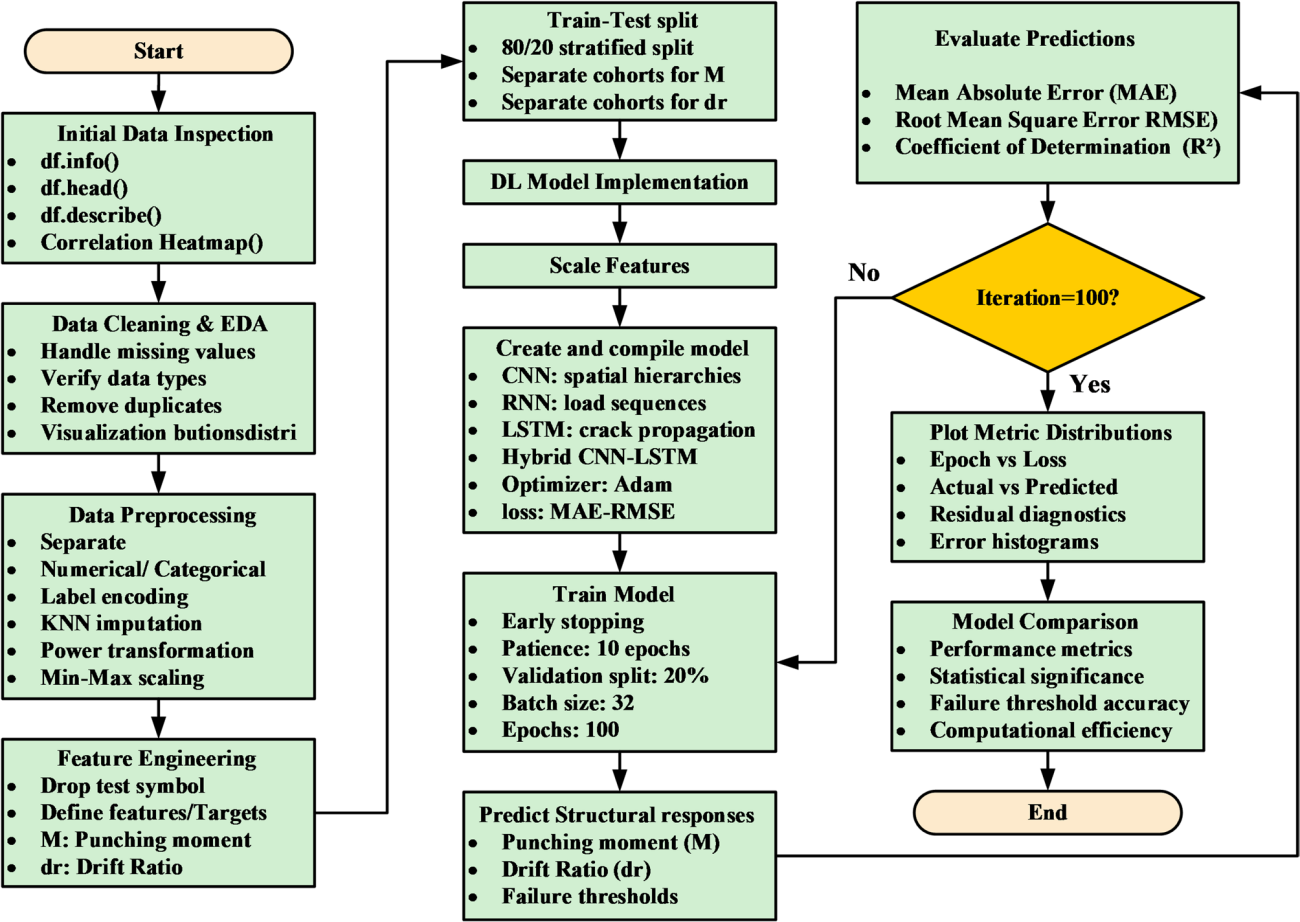
Sequential DL framework for predicting (M and dr) in RC systems

**Fig. 9:**
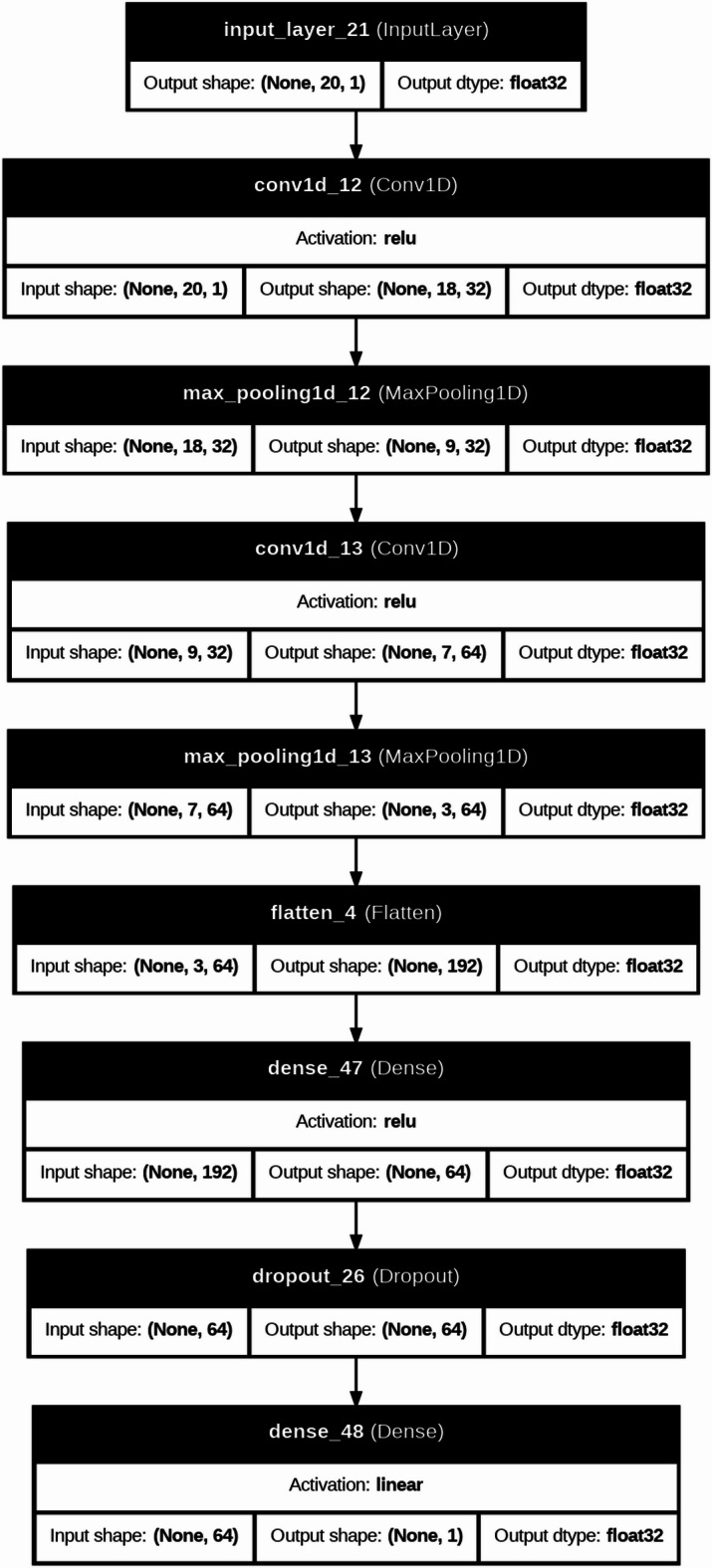
Architecture of the CNN model is used for spatial feature extraction from structural data

**Fig. 10:**
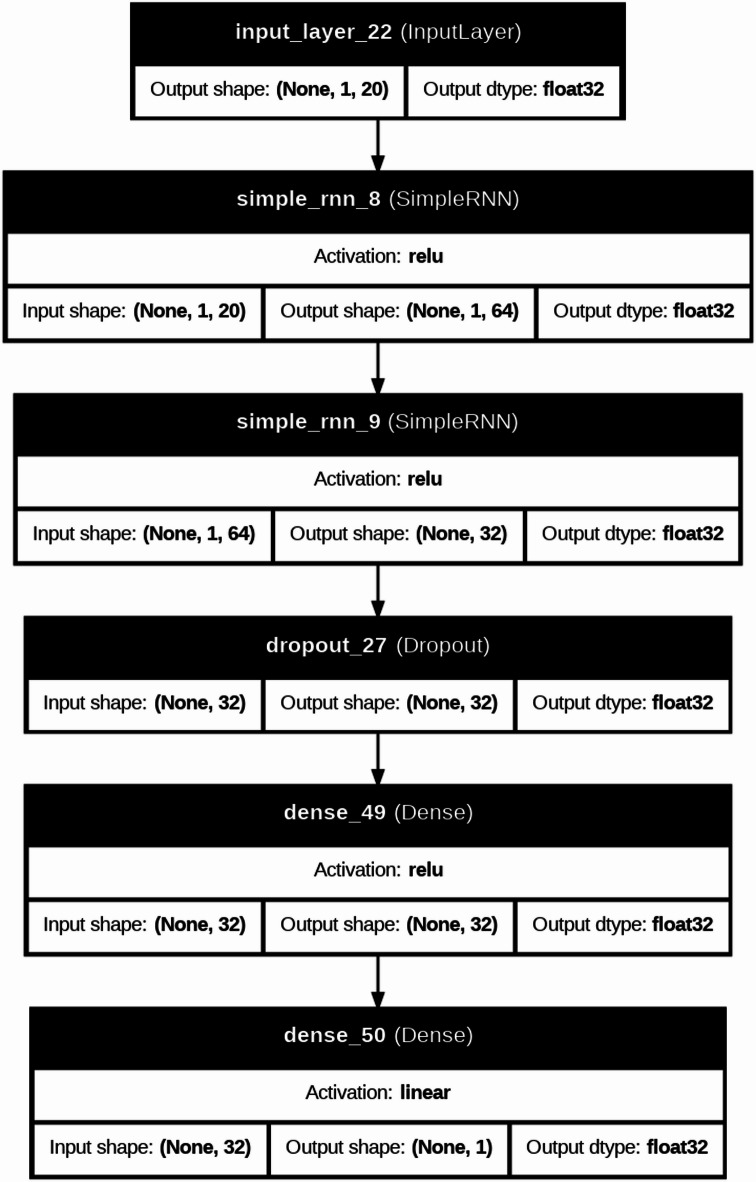
Architecture of the RNN model for modeling sequential structural response

**Fig. 11:**
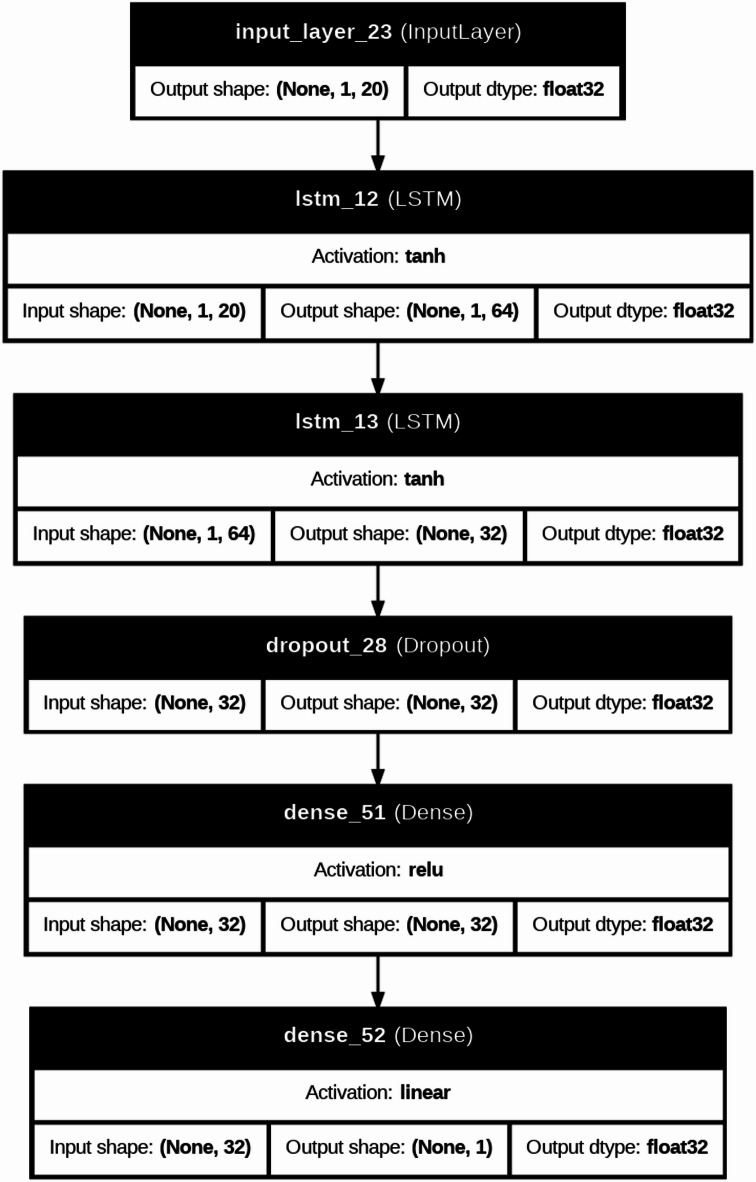
Architecture of the LSTM network for capturing long-term dependencies

**Fig. 12:**
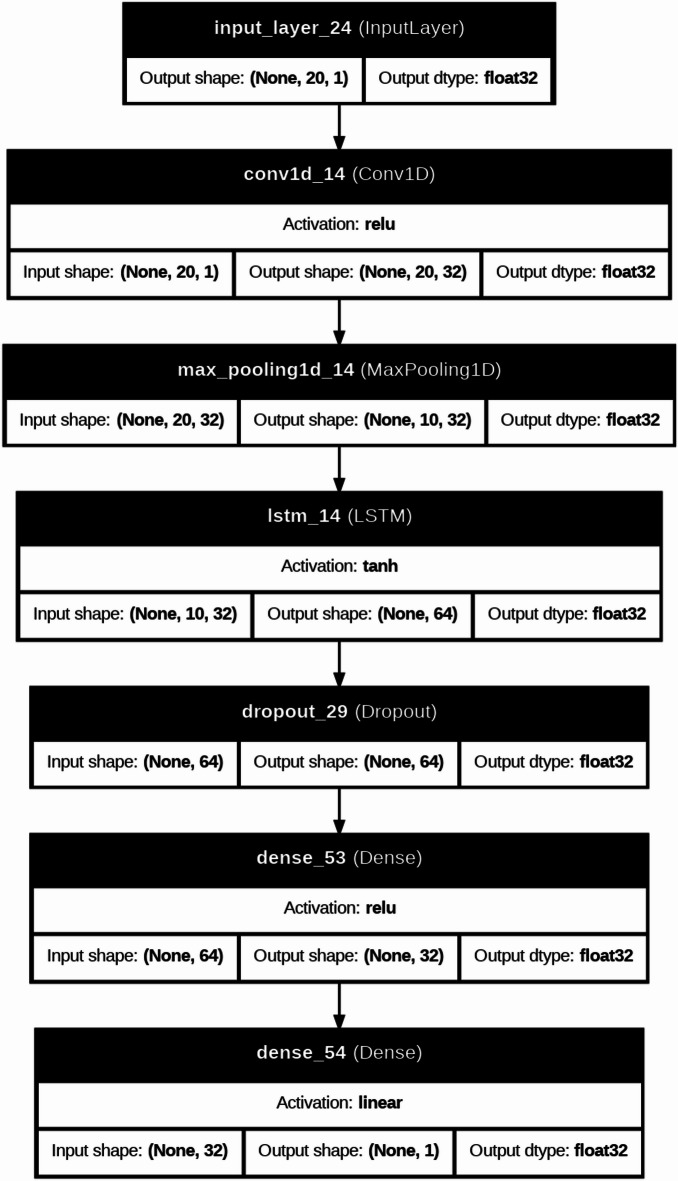
A hybrid CNN-LSTM architecture combining spatial and temporal feature modeling

DL models are typically applied to large-scale datasets or image-based problems; they are included in this study to investigate their capability in capturing complex nonlinear relationships within structured engineering data. In this context, DL architectures such as CNN, RNN, and LSTM are evaluated to assess whether their representation learning ability can provide additional predictive value compared to traditional ML models. Furthermore, the CNN-LSTM model is explored as a hybrid approach that combines feature extraction capabilities of convolutional layers with the interaction learning potential of LSTM units, aiming to capture complex dependencies among input variables^42–45^. The architectures of DL were used to predict severe structural failure processes like (M and dr) in RC systems. In the present study, the DL models were not fed with image-based or time-series data; instead, they were trained using structured tabular input. Each specimen was represented by a one-dimensional feature vector containing the geometric, material, reinforcement, and loading variables. For implementation in the CNN, RNN, and LSTM models, this vector was reshaped into an ordered one-dimensional input sequence, where each feature was treated as one input step/channel in the network. Therefore, the DL models were used to learn inter-feature dependencies within the tabular dataset rather than true spatial or temporal patterns in the conventional sense. This is a computationally intensive model, which follows iterative refinement cycles and uses the adaptive learning properties of neural networks to convert raw structural data into predictive information used in the detection of a collapse. Figure 8 is a more detailed description of our DL model for forecasting these important structural failure mechanisms in the RC systems. This is a computationally heavy procedure in which the adaptive learning abilities of neural networks are utilized via refinement cycles to improve dataset refinement through a carefully executed dataset preparation stage, where extra metadata (test identifiers) has been removed to maximize the relevance of features. Correlation heatmaps are a visual form of interrogative relationship that simplifies querying the relationship between geometric factors (slab dimensions, column sizes), material (concrete strength, relationship between reinforcement and material), and loading features, and simplifies feature selection to be further used in the model. The data is then partitioned into a training and test set, and distributional normalization is done using power transformations and min-max scaling to ensure stability in convergence. Four architectures are then built, each focused on the tasks of spatial stress field in slab column joints, CNN models, time variable loads development CNN, RNN models, crack development pattern, and a hybrid CNN LSTM architecture that combines hierarchies in space with time deformation evolution. Both models have task-specific activation functions (ReLU and tanh) and Adam optimizer, and during training include early stop procedures of 100 or more epochs to stop overfitting and adjust weights backpropagationally as structural responses are predicted and evaluated during training against experimental failure limits. Evaluations on post-training performance are measured by monitoring the R^2^, MAE, and RMSE performance indicators over the epochs, and the error plots ascertained that the measurements remain stable throughout, as the shear failure response is seen to be recorded. Table 3 compared the performance of these architectures relatively and found that the hybrid CNN LSTM architecture has a better performance in the nonlinear interaction between reinforcement layouts and deformation limits. To further elaborate how both models work, Fig. 7 gives a structural composition of the architectures used in the computational flow of each of the architectures in Figs. 9, 10, 11 and 12. Figure 9 introduces the CNN architecture, and this architecture consists of consecutive convolutional layers that carry with them localized filters to scan the input data and capture geometrical features such as the area of stress concentration or geometric discontinuities in slab column joints. These characteristics are then narrowed down in dimensionality by means of pooling layers, which purvey prevailing information and enhance computational efficiency. This can be further fed directly to one or more fully connected layers, which teach higher-order interactions and produce the final prediction. Figure 10 presents the structure of the RNN, which is specifically developed to deal with sequential data. In this form, the network is in a hidden state, which is updated in an iterative manner by adding new inputs with the memory of past time steps; therefore, the evolving loading patterns or time-dependent material behavior can be modeled. Nevertheless, vanishing gradients frequently tend to make the RNNs susceptible, limiting their capacity to memorize long-term dependencies. To this end, the LSTM network in Fig. 11 builds upon the recurring architecture by including gated memory units, which control the flow of information through an input, forget and output gate which decides which information is retained, discarded or forwards to the next time step, and thus the network to sustain applicable long-term dependencies, including the propagation of cracks or dropped out deformations under cyclical loads. Figure 12 shows the hybrid CNN LSTM model that combines the ability of CNN in extracting spatial features and the ability of LSTM in modeling cases over time. This architecture uses convolutional and pooling layers to first encode non-spatial structural data in meaningful features of space. The processed features are then reworked and input into LSTM layers, learning the temporal development of the said features, e.g., progressive movement or redistribution of stress. This combined model is a well-rounded expression of the structural behavior as it includes such issues as the spatial correlations as well as time-dependent responses, which is an important variable in the accurate depiction of the complicated failure mechanisms of RC in dynamic and nonlinear scenarios.

**Table 3:** Comparative analysis of DL algorithms for structural performance prediction

Algorithm	Primary use	Complexity	Advantages	Disadvantages	Interpretability
CNN	Spatial feature extraction in structural joints	High	Detects localized stress patterns 32 filters capture critical features Optimal kernel_size = 3 for geometry recognition	Requires spatial structuring of input data Sensitive to kernel size selection	Low
RNN	Sequential load progression modeling	High	Manages time-dependent loading sequences 64 units balance complexity/performance Effective for cyclic load patterns	Struggles with long-term dependencies Vanishing gradient issues	Low
LSTM	Long-term deformation tracking	Very High	Captures crack propagation sequences Handles 50-epoch training efficiently Patience = 5 prevents overfitting	Computationally intensive Batch size = 32 requires memory optimization	Low
Hybrid CNN-LSTM	Integrated spatiotemporal failure analysis	Very High	Combines CNN’s spatial detection (filters = 32) with LSTM’s temporal tracking Optimal for combined geometry/loading analysis	Extremely resource-intensive Complex hyperparameter tuning	Very Low

### Experimental setup

The Python programming language was used for the implementation of the proposed ML and DL models. For testing purposes, a Google Collaboratory (Colab) Linux server with Ubuntu 16.04 was used for all experimental testing. This free cloud-based service provides hardware options like Central Processing Unit (CPU), Tesla K80 Graphics Processing Unit (GPU), and Tensor Processing Unit (TPU).

### Performance metrics

To strictly assess the predictive power of the models, this paper adopted a set of appropriate statistical measures. All measures were chosen with care, in the sense that they provide complementary insights into model performance: its capability to explain structural behavior, to quantify errors of magnitude, and to describe the statistical nature of predictive discrepancies. The extent of the linear relation between the predicted and actual structural responses was measured by the first metric, R^2^, which is computed as the ratio obtained by the summation of the products of deviations of the predicted and observed value of means and product of their standard deviations as given in Eq. 1^46,47^. The second measure, MAE, is used to determine the average error rate between actual experimental values and the predictive results. This measure, as explained by Eq. 2^48,49^, is a simple method of quantifying the average size of error that a model generates, without consideration of the fact that the model may be overestimating or underestimating the true outcomes. The third evaluation criterion, RMSE, is used to supplement MAE because it focuses on the effect of greater errors. It is determined by squaring both the error in predictions and the average of the squared values, and then taking the square root of the value, Eq. 3 below^50,51^. RMSE is especially useful in pointing out the sensitivity of the model to large outliers, which can be present in very nonlinear structural behavior. The mathematical expression of these measures is as follows:

1 $${\mathrm{R}}^{2}=1-\frac{\sum_{i=1}^{N}({y}_{i}-{\widehat{y}}_{i}{)}^{2}}{\sum_{i=1}^{N}({y}_{i}-\stackrel{\prime }{y}{)}^{2}}$$

2 $$\mathrm{MAE}=\frac{1}{n}\sum\limits_{i=1}^{n}\mid {y}_{i}-{\widehat{y}}_{i}\mid$$

3 $$\mathrm{RMSE}=\sqrt{\frac{1}{n}\sum\nolimits_{i=1}^{n}({y}_{i}-{\widehat{y}}_{i}{)}^{2}}$$

where ($\:{y}_{i}$) is the actual value, ($\:{\widehat{y}}_{i}$) is the predicted value, ($\:\stackrel{\prime }{y}$) is the mean of the observed values and ($\:n$) is the number of datapoints.

Combining these three complementary evaluation measures, this research develops a strong and effective model for measuring model performance. In conventional ML, the models were trained and evaluated only once, though in the case of the DL models, the models were trained over 100 epochs, and the early stop policies were used to guarantee the best convergence and generalization. This assessment plan provides a significant comparison between the architectures and helps to choose the most reliable models to predict the punching shear failure and drift behavior of RC structures correctly.

## Results and discussion

Following the results offered in this paper, the predictive accuracy of both ML and DL models was tested properly within the framework of predicting structural failure parameters in RC systems, namely, M and dr. In order to have the complete consideration of the evaluation, the study employs three basic performance measures: R^2^, MAE, and RMSE. These measures collectively give a balanced evaluation, in the sense of linear agreement between model predictions and observations, of mean prediction error, and sensitivity of the model to large deviations. Such a combination allows further insight into the reliability and the strength of each of the models in the simulation of the intricate structural behavior under different loading conditions.

### ML models’ performance

Adopting the comparative analysis of ML models, a specialized analysis was created to evaluate their prediction capabilities in the estimation of two structural indicators that are important in RC systems (M and dr). These analyses were undertaken based on three basic statistical measures (R^2^, MAE, RMSE) as shown in Tables 4 and 5. In (M)prediction as in Table 3, the traditional linear models, such as LR, RR, and Lasso R, had low predictive accuracy as indicated by lower values of R^2^, including 0.745, and large values of errors. These findings point to the limited capacity of linear algorithms to model very complicated and nonlinear relationships between structural behavior. It was found that there were significant increases in prediction accuracy as the model complexity was increased. The generalization of SVR was also superior to that of linear models, as it had a higher R^2^ of 0.797 and smaller error measures. The highest gains were given by ensemble-based models. RF and GB showed strong performance, with GB having an R^2^ value of 0.870, an RMSE value of 0.378, and an MAE value of 0.228. The XGBoost model was the best performing with a maximum R^2^ value of 0.875, a minimum MAE of 0.313, and an RMSE of 0.397. The GB and XGBoost models are especially remarkable because they have the best correlation and reduced error rates that prove their great capability of identifying sophisticated structural patterns. Conversely, Table 5 discussed the performance of the ML models in predicting the dr showed a higher variation and lesser predictive ability. The weakest results were once again obtained by the models based on linear and LR, with a low R^2^ of 0.36 and the greatest error rates, respectively. RR and Lasso R have slightly improved, but their predictive accuracy was low. Linear models also had significant superiority over SVR in this task. RF was found to be the best algorithm to predict drift as it achieved the highest R^2^ of 0.565 and the lowest RMSE of 0.619, which showed a better compromise between bias and variance in modeling the drift behavior. The same dominance was not observed in ensemble methods like GB or XGBoost on the (M) task, which could indicate that the dr prediction problem could be having other underlying patterns that are not well represented by such algorithms. This fact makes it clear that model selection should be matched with the character of the structural parameter under prediction.

**Table 4:** Comparative performance metrics of ML algorithms for predicting (M) in RC systems

Algorithm	*R* ^2^	MAE	RMSE
LR	0.743129	0.385684	0.533440
RR	0.741158	0.384038	0.535482
Lasso R	0.740044	0.392499	0.536633
EN	0.740050	0.391664	0.536627
SVR	0.797212	0.343247	0.473968
RF	0.840906	0.319026	0.419812
GB	0.870850	0.282686	0.378246
XGBoost	0.857051	0.313696	0.397941

**Table 5:** Performance comparison of ML algorithms for predicting dr in RC structures

Algorithm	*R* ^2^	MAE	RMSE
LR	0.363381	0.563724	0.749425
RR	0.457511	0.535020	0.691805
Lasso R	0.458628	0.531315	0.691093
EN	0.471096	0.537920	0.683088
SVR	0.435218	0.490301	0.705877
RF	0.565014	0.440262	0.619478
GB	0.390692	0.520714	0.733174
XGBoost	0.438491	0.502796	0.703828

### DL models’ performance

In this study, a DL model was used, where the hyperparameters were optimized with high accuracy in predicting electrochemical currents, and fully presented in Table 6. The model architecture was used in the form of 64 units, 32 filters, a kernel size of 3, a dropout rate of 0.2, and the ReLU activation function. Training was done using Adam optimizer with a learning rate of 0.001 and a batch size of 32. There were 50 epochs, and early stopping (patience = 5) was applied to minimize overfitting and enhance generalization. This framework was used to conduct a comparative evaluation between four DL models, i.e., CNN, RNN, LSTM, and CNN-LSTM, in the predictive action of the structural response of RC systems. Three standard performance measures were put into the evaluation ( R^2^, MAE, RMSE). Table 7 demonstrates that the CNN model had the highest accuracy in forecasting the M with a value of R^2^ = 0.827864, a MAE = 0.335228, and a RMSE = 0.436680. The RNN model was second in predictive performance, with the LSTM and the CNN-LSTM models having much less accuracy. CNN-LSTM has the lowest results with an R^2^ of 0.702617, an MAE of 0.466479, and an RMSE of 0.573965. These findings indicate that the complexity of the hybrid model might not be warranted based on the character of the input data, which might not possess time dependencies. In the same way, the CNN model outperformed the others in obtaining an R^2^ of 0.470568, an MAE of 0.513140, and an RMSE of 0.683429 in the case of the dr prediction in Table 8. The generalized accuracy of prediction was, however, poorer with dr than M in all models. The CNN- LSTM model had the worst results with R^2^ of 0.297733, MAE of 0.656307, and RMSE of 0.787117, and thus it is not very effective in this task. The results in Tables 7 and 8 help to compare the distribution of error and trends in prediction among the tested models, highlighting the ability of CNN to show consistency in prediction when other models, especially traditional approaches of ML, show significant deviations. The relative stability of CNN on different structural responses adds to the fact that CNN is a reliable predictive model in any engineering context. In general, the general outperformance of the CNN model in both (M and dr) prediction tasks demonstrates its usefulness in problems of structural engineering, where the accuracy of forecasting is an essential criterion. Conversely, the consistent poor performance of the CNN-LSTM hybrid model highlights the role of model architecture in terms of ensuring that the model is adjusted to the underlying data properties to deliver the best outcomes.

**Table 6:** Core hyperparameters and model settings used for electrochemical current prediction

Parameter	Value
Number of units	64
Number of filters	32
Kernel size	3
Dropout rate	0.2
Activation function	ReLU
Optimizer	Adam
Base learning rate	0.001
Batch size	32
Training Epochs	50
Early stopping patience	5 epochs

**Table 7:** Comparative performance metrics of DL algorithms for predicting (M) in RC systems

Algorithm	*R* ^2^	MAE	RMSE
CNN	0.827864	0.335228	0.436680
RNN	0.779650	0.369975	0.494065
LSTM	0.749241	0.410441	0.527055
CNN_LSTM	0.702617	0.466479	0.573965

**Table 8:** Comparative performance metrics of DL algorithms for predicting dr in RC structures

Algorithm	*R* ^2^	MAE	RMSE
CNN	0.470568	0.513140	0.683429
RNN	0.355629	0.560308	0.753974
LSTM	0.423241	0.561079	0.713322
CNN-LSTM	0.297733	0.656307	0.787117

### Comparative insights

This behavior can be interpreted from a structural engineering perspective, where the prediction of (M) depends on complex localized nonlinear interactions between geometric and material properties, which are effectively captured by boosting-based models such as GB. In contrast, dr represents a global deformation response influenced by multiple distributed parameters, where ensemble averaging approaches such as RF provide more robust and stable predictions. These differences highlight that model performance is strongly linked to the physical nature of the structural response being predicted. The superiority is well manifested in the R^2^ that reveals the higher levels of linear correlation between predicted and actual values, as well as significantly lower levels of error measures, like MAE and RMSE. The results indicate that ML models are more reliable and capable of predicting structural behavior of (M and dr) since they are flexible to deal with the limited data and require less computational complexity than the DL models. Tables 4, 5, 6, 7, and 8 present the critical evaluation of the predictive capacities of the ML and DL models in the determination of the (M and dr) of the RC structures. The findings demonstrate the higher performance of the ML models as compared to the DL counterparts in this field. This superiority is well manifested in the R^2^ that reveals the higher levels of linear correlation between predicted and actual values, as well as significantly lower levels of error measures, like MAE and RMSE. The results indicate that ML models are more reliable and capable of predicting structural behavior of (M and dr) since they are flexible to deal with the limited data and require less computational complexity than the DL models.

#### Data scale and complexity

ML models can perform more confident predictions in cases where the variables are related simply or when the size of the dataset is small. They are especially applicable in engineering applications that do not have as much extensive experimental data due to their performance on small sets of data. Conversely, DL models generally need extensive amounts of data to be able to reflect complicated and nonlinear trends successfully. In the absence of enough data, their performance benefit is likely to decrease, and they do not necessarily perform much better than the traditional ML methods, particularly on structural engineering data with parameters such as (M and dr).

#### Feature design and model explainability

The feature engineering is the process that allows enhancing the accuracy and clarity of the predictions of the ML models. ML models can also more easily identify meaningful patterns in the data because they select and transform input variables, guided by domain knowledge. As compared to this, DL models naturally extract features using raw data, which in numerous instances can be beneficial. Nonetheless, this automatic solution could prove to be ineffective when the predictive task presents a delicate or a very specific relationship to be brought out, which can be better emphasized by explicit feature design, especially in engineering, where the physical parameter is well-defined.

#### Computational efficiency

As opposed to the DL models, the ML models are lightweight in terms of computational needs and time to train. This renders them an effective option in contexts where resources are scarce or where the fast development and deployment of the model is of utmost importance. Their reduced complexity and accelerated processing further imply that the ML methods are particularly attractive in the context of real-time applications or when the hardware setup is relatively small.

#### Model complexity and predictive behavior in RC systems

**Fig. 13:**
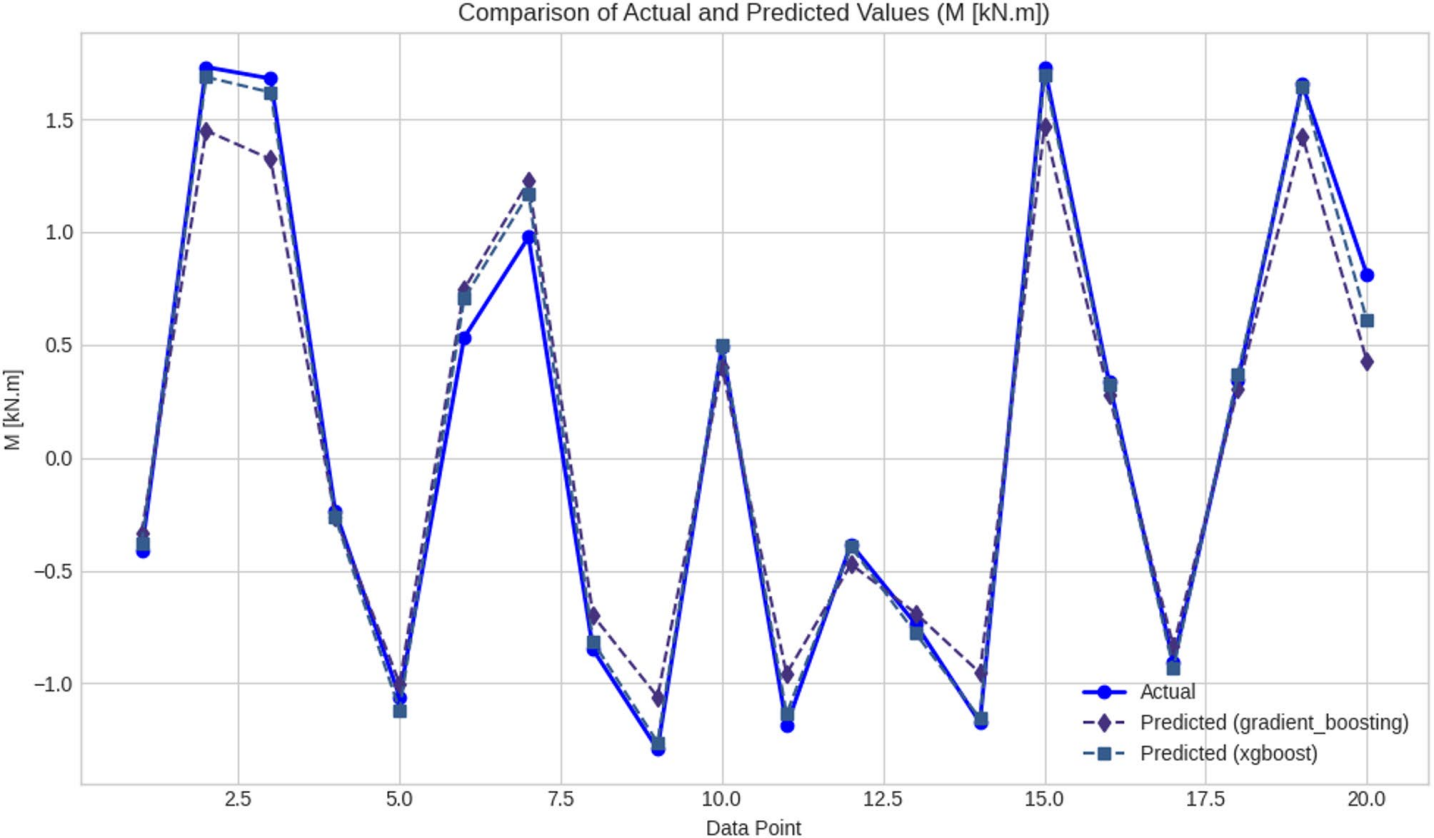
Comparison of (M) predictions from GB and XGBoost models across a 20-point dataset

**Fig. 14:**
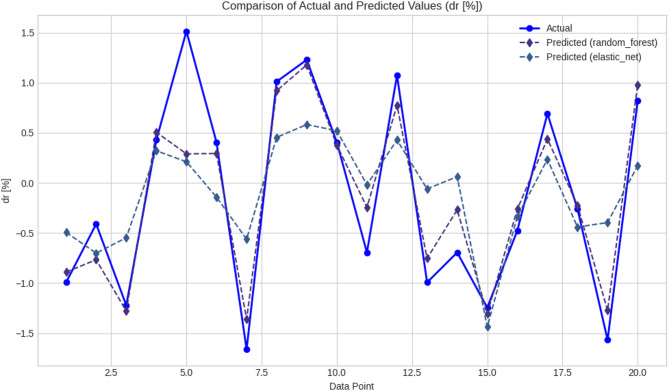
Evaluation of RF and EN model performance for dr (%) predictions on a 20-point dataset

Overall analysis of the performance of models showed that there were pronounced differences between the ML and DL models when used to predict (M and dr) in RC elements. Tables 4 and 5 give quantitative theoretical evidence that tree-based ML models, specifically GB, XGBoost, and RF, were more effective in terms of accuracy and error reduction than both linear regressors and DL options. The GB model performed best in terms of the overall performance of any of the ML algorithms, with R^2^ showing the best value of 0.870850, MAE of 0.282686, and RMSE of 0.378246 in prediction (M). XGBoost came next with a marginally greater MAE and RMSE values, but with a good R^2^ value of 0.857051. These two models, as observed in Table 5 and as further shown in Table 9; Fig. 13, gave high levels of sensitivity in predicting the actual structural responses and had low levels of residual errors in predicting the actual responses in the dataset. This high consensus implies that ensemble tree models are specifically created to deal with nonlinear dependencies in tabular engineering data. Conversely, the DLs of Table 7, such as CNN, RNN, LSTM, and CNN-LSTM, had relatively lower predictive performance in terms of estimating (M). Even though CNN recorded a reasonable value of R^2^ of 0.827864, the MAE and RMSE of CNN were higher than those of GB and XGBoost. Other architectures, including RNN and CNN-LSTM, had even lower accuracy and higher prediction variability, which implies that they could learn the structural dependencies in the data. RF was superior to the rest of the ML algorithms in terms of dr prediction, as Table 5 confirmed that RF had the highest R^2^ at 0.565014, the highest MAE at 0.440262, and RMSE at 0.619478. EN was less erroneous than other models but could not reproduce nonlinear tendencies because it is formulated in a linear format. Such a difference is well-seen in Table 10; Fig. 14, where RF gave more accurate and consistent forecasts than EN, especially in the data points with higher absolute values. Table 8 demonstrates that TV- DL models did not do well in forecasting the dr. R^2^ values of CNN, RNN, and LSTM fell between 0.297 and 0.470, and the MAE and the RMSE were high. This performance difference indicates that DL would be less appropriate in small to medium-sized structured data, where the relationship between features is not hierarchically structured, as is the case with neural networks. All these findings collectively support the feasibility and performance in relation to tree-based algorithms of ML models in the prediction of important structural performance levels, including (M and dr). These aptitudes on how to handle the different feature interactions at minimal computational costs make them very effective tools for modeling structural behavior. The results highlight the necessity of matching the complexity of models with the characteristics of data and prediction tasks since less sophisticated but more effective algorithms, such as GB and RF, provided more trustworthy and comprehensible results than more sophisticated DL architectures. The evaluation of the proposed models was not restricted to statistical measures but was also assessed from an engineering perspective. In this regard, the values of the punching moment (M) and drift ratio (dr), as predicted in the proposed models and shown in Tables 9 and 10, were evaluated to ensure that they fall within structurally acceptable ranges and satisfy the expected trends of the response of the slab-column connections under seismic loading. In this regard, the trends of the proposed models were found to align with the influence of some of the major governing parameters, including the slab thickness, column dimensions, material properties, and reinforcement properties. From an engineering perspective, it can thus be deduced that although the best-performing ML models can serve as adequate support for the seismic assessment of structures, they should not be used in isolation.

**Table 9:** Evaluation of GB and XGBoost models: actual versus predicted (M) values

Data point	Actual M (kN m)	Predicted (GB)	Predicted (XGBoost)
1	−0.4135	−0.3320	−0.3794
2	1.7313	1.4493	1.6870
3	1.6800	1.3225	1.6191
4	−0.2348	−0.2609	−0.2621
5	−1.0590	−1.0009	−1.1185
6	0.5310	0.7458	0.7047
7	0.9775	1.2257	1.1701
8	−0.8499	−0.6993	−0.8157
9	−1.2920	−1.0597	−1.2623
10	0.4919	0.4008	0.5018
11	−1.1883	−0.9577	−1.1356
12	−0.3830	−0.4719	−0.3945
13	−0.7360	−0.6884	−0.7772
14	−1.1732	−0.9538	−1.1561
15	1.7313	1.4672	1.6951
16	0.3378	0.2793	0.3217
17	−0.9065	−0.8357	−0.9345
18	0.3422	0.3040	0.3707
19	1.6538	1.4249	1.6412
20	0.8116	0.4279	0.6078

**Table 10:** Comparison of actual and predicted dr (%) values using RF and EN models

Data point	Actual dr (%)	Predicted (RF)	Predicted (EN)
1	−0.9933	−0.8898	−0.4936
2	−0.4103	−0.7672	−0.7011
3	−1.2226	−1.2790	−0.5474
4	0.4276	0.5056	0.3202
5	1.5138	0.2897	0.2111
6	0.4050	0.2929	−0.1422
7	−1.6626	−1.3601	−0.5618
8	1.0106	0.9197	0.4543
9	1.2285	1.1785	0.5825
10	0.4050	0.3759	0.5204
11	−0.6944	−0.2434	−0.0185
12	1.0733	0.7743	0.4279
13	−0.9933	−0.7539	−0.0601
14	−0.6944	−0.2684	0.0619
15	−1.2458	−1.3065	−1.4378
16	−0.4753	−0.2617	−0.3349
17	0.6908	0.4390	0.2350
18	−0.2602	−0.2242	−0.4408
19	−1.5670	−1.2734	−0.3957
20	0.8199	0.9790	0.1718

## Conclusion

This paper presents a detailed comparative analysis of the two models (ML and DL) of predicting key structural performance variables, namely, M and dr, in RC systems. In a range of models, the findings show that the prediction capabilities of ML algorithms, especially those based on trees like GB, XGBoost, and RF, are better. In the case of (M) prediction, GB achieved its highest accuracy with (R^2^=0.870850, MAE = 0.282686, and RMSE = 0.378246), followed by close and strong scores of XGBoost and RF. In the case of dr prediction, RF achieved optimal results (R^2^ = 0.565014, MAE = 0.440262, RMSE = 0.619478), which highlights the strengths of ML methods in the prediction of nonlinear relationships in structured engineering data. On the other hand, DL models demonstrated relatively worse predictive performance in the identical settings, and this demonstrates that they are sensitive to the amount and structure of data. The CNN architecture achieved the best result in M prediction (R^2^ = 0.827864), and LSTM and RNN have moderate performance, and the hybrid CNN to LSTM model failed to solve the dr prediction (R^2^ = 0.297733). However, the hybrid approach demonstrated the possibilities of mixing the spatial and sequential learning of features to improve the level of prediction fidelity, especially with the assistance of enhanced data preprocessing and feature engineering. These results attest to the fact that model selection is not only a technical process but also a strategic process, which ultimately determines the reliability, interpretability, and generalizability of predictive information in structural engineering. The established outstanding efficiency of ML approaches to structured tabular data indicates their pragmatism, whereas the exploration application of hybrid DL models represents a positive perspective of addressing more complex data patterns. In the future, this study provides the foundation of smart and data science-driven models capable of aiding structural assessment, monitoring, and design. The next step will be to increase the accuracy and interpretability of prediction by using attention mechanisms, feature fusion strategies, and superior hybrid modeling models that integrate ML and DL teams. Future work will also incorporate additional parameters that influence punching shear failure, including shear ratio, P-delta effects, and connection details, to further enhance the predictive accuracy of the models.

## Data Availability

The datasets used and/or analyzed during the current study are available from the corresponding author on reasonable request. You can contact Mahmoud A. El-Mandouh in case of requesting study data. this email: [m.abdel.aziz.mohsen@gmail.com](mailto: m.abdel.aziz.mohsen@gmail.com).

## **Abbreviations**

AI: Artificial Intelligence
CNN: Convolutional Neural Network
DL: Deep Learning
DNN: Deep Neural Network
dr: Drift Ratio
EN: Elastic Net
FRP: Fiber Reinforced Polymer
GB: Gradient Boosting
Lasso R: Lasso Regression
LR: Linear Regression
LSTM: Long Short-Term Memory
M: Punching Moment
MAE: Mean Absolute Error
MIDR: Maximum Interstory Drift Ratio
ML: Machine Learning
PHL: Plastic Hinge Length
R^2^: Coefficient of Determination
RC: Reinforced Concrete
RF: Random Forest
RMSE: Root Mean Square Error
RNN: Recurrent Neural Network
RR: Ridge Regression
SVR: Support Vector Regression
XGBoost: Extreme Gradient Boosting
